# Oral Migalastat HCl Leads to Greater Systemic Exposure and Tissue Levels of Active α-Galactosidase A in Fabry Patients when Co-Administered with Infused Agalsidase

**DOI:** 10.1371/journal.pone.0134341

**Published:** 2015-08-07

**Authors:** David G. Warnock, Daniel G. Bichet, Myrl Holida, Ozlem Goker-Alpan, Kathy Nicholls, Mark Thomas, Francois Eyskens, Suma Shankar, Mathews Adera, Sheela Sitaraman, Richie Khanna, John J. Flanagan, Brandon A. Wustman, Jay Barth, Carrolee Barlow, Kenneth J. Valenzano, David J. Lockhart, Pol Boudes, Franklin K. Johnson

**Affiliations:** 1 University of Alabama at Birmingham School of Medicine Department of Medicine, Division of Nephrology, Birmingham, Alabama, United States of America; 2 University of Montreal, Quebec, Canada; 3 University of Iowa Children’s Hospital, Iowa City, Iowa, United States of America; 4 LSD Research and Treatment Unit, O&O Alpan LLC, Fairfax, Virginia, United States of America; 5 Department of Nephrology, Royal Melbourne Hospital, and University of Melbourne, Parkville, Victoria, Australia; 6 Linear Clinical Research Ltd, Queen Elizabeth II Medical Centre, and University of Western Australia, Nedlands, Western Australia; 7 Universitair Ziekenhuis Antwerpen, Antwerpen, Belgium; 8 Emory University School of Medicine, Emory Genetics Clinic, Athens, Georgia, United States of America; 9 Amicus Therapeutics, Cranbury, New Jersey, United States of America; 10 Arvinas Inc, New Haven, Connecticut, United States of America; 11 Institute and Clinical Center, Sunnyvale, California, United States of America; 12 Cymabay Therapeutics, Newark, California, United States of America; University of Manchester, UNITED KINGDOM

## Abstract

Migalastat HCl (AT1001, 1-Deoxygalactonojirimycin) is an investigational pharmacological chaperone for the treatment of α-galactosidase A (α-Gal A) deficiency, which leads to Fabry disease, an X-linked, lysosomal storage disorder. The currently approved, biologics-based therapy for Fabry disease is enzyme replacement therapy (ERT) with either agalsidase alfa (Replagal) or agalsidase beta (Fabrazyme). Based on preclinical data, migalastat HCl in combination with agalsidase is expected to result in the pharmacokinetic (PK) enhancement of agalsidase in plasma by increasing the systemic exposure of active agalsidase, thereby leading to increased cellular levels in disease-relevant tissues. This Phase 2a study design consisted of an open-label, fixed-treatment sequence that evaluated the effects of single oral doses of 150 mg or 450 mg migalastat HCl on the PK and tissue levels of intravenously infused agalsidase (0.2, 0.5, or 1.0 mg/kg) in male Fabry patients. As expected, intravenous administration of agalsidase alone resulted in increased α-Gal A activity in plasma, skin, and peripheral blood mononuclear cells (PBMCs) compared to baseline. Following co-administration of migalastat HCl and agalsidase, α-Gal A activity in plasma was further significantly increased 1.2- to 5.1-fold compared to agalsidase administration alone, in 22 of 23 patients (95.6%). Importantly, similar increases in skin and PBMC α-Gal A activity were seen following co-administration of migalastat HCl and agalsidase. The effects were not related to the administered migalastat HCl dose, as the 150 mg dose of migalastat HCl increased α-Gal A activity to the same extent as the 450 mg dose. Conversely, agalsidase had no effect on the plasma PK of migalastat. No migalastat HCl-related adverse events or drug-related tolerability issues were identified.

**Trial Registration:**

ClinicalTrials.gov NCT01196871

## Introduction

Fabry disease is an X-linked lysosomal storage disorder (LSD) caused by inherited mutations in the gene (*GLA*) that encodes α-galactosidase A (α-Gal A).[1,2] Deficiency of α-Gal A results in progressive accumulation and deposition of neutral glycosphingolipids with terminal α-galactosyl residues, primarily globotriaosylceramide (GL-3, also known as Gb3 or CTH [ceramide trihexoside]), in cells of the heart, kidney, skin, brain, and other tissues, which is believed to contribute to the life-threatening manifestations of Fabry disease.[1–3] The clinical presentation of Fabry disease spans a broad spectrum of severity and roughly correlates with residual α-Gal A activity.[2,4]

Enzyme replacement therapy (ERT) is currently the primary treatment for Fabry disease. ERT is based on the intravenous administration of manufactured human α-Gal A, of which Fabrazyme (agalsidase beta; Genzyme, Cambridge, MA) and Replagal (agalsidase alfa; Shire Pharmaceuticals, Cambridge, MA) are the only two approved products. These therapies have been shown to reduce plasma, urine, and capillary endothelial GL-3 levels, [5,6] stabilize kidney function, [7] and alleviate neuropathic pain, and reverse or improve hypertrophic cardiomyopathy. [6,8,9] However, the infused enzymes tend to be unstable at neutral pH of blood, resulting in a short-circulating half-life of the properly folded active enzyme *in vivo*. [5,10] Furthermore, delivery and uptake of ERT to some cell types is insufficient in certain cases, as suggested by the inability of infused enzyme to significantly reduce GL-3 in cardiomyocytes, distal convoluted tubules, and glomerular podocytes, as well as the central nervous system. [5,10] In addition, the infused enzymes can be immunogenic, which may limit efficacy [11] and may adversely affect tolerability. [12,13] Current guidelines do not distinguish between the use of agalsidase beta and agalsidase alfa.

Small molecule pharmacological chaperones (PCs) have been proposed as a potential therapy for Fabry disease. [14] The iminosugar, 1-deoxygalactonojirimycin (AT1001, hereafter referred to as migalastat hydrochloride (HCl)) is an analog of the terminal galactose of GL-3 that selectively and reversibly binds and stabilizes wild-type and some mutant forms of α-Gal A, [15,16] and when used alone as a monotherapy has been shown to reduce the storage of GL-3 *in vitro* and *in vivo*. [17–20] In contrast to ERT, migalastat HCl is orally available and has broad tissue distribution. [19] As such, migalastat HCl is currently in clinical development to evaluate its safety and efficacy as a monotherapy for Fabry disease. [21] PCs have also been identified that selectively bind and increase the levels of mutated enzymes associated with several other LSDs, including Gaucher, Tay-Sachs, Sandhoff, GM1-gangliosidosis, and Pompe disease. [22]

Migalastat HCl was well tolerated in healthy volunteers after single doses up to 2000 mg and after multiple daily doses up to 300 mg administered as 150 mg twice a day (BID), and in Fabry patients at daily doses up to 500 mg, administered as 250 mg BID. The plasma and urine pharmacokinetics of migalastat were well characterized in nine Phase 1 studies conducted in healthy volunteers. [23] One-hundred-fifty milligrams administered every other day was evaluated in Phase 2 studies and is currently being evaluated in Phase 3 studies as a single agent for the treatment of Fabry disease in patients with *GLA* mutations that are amenable to chaperone therapy.

The clinical rationale for this study was to provide drug-drug interaction information after co-administration of migalastat HCl with agalsidase. In addition, information on the effect of migalastat HCl on agalsidase was obtained for proof of concept that migalastat HCl has the potential to improve the pharmacokinetic properties of agalsidase in Fabry patients.

Two dose levels of migalastat HCl were evaluated in this study, 150 mg and 450 mg. The 150 mg dose was selected based upon statistically significant clinical outcomes in the Fabry monotherapy program in Fabry patients with amenable mutations, and a 3X dose was selected to evaluate any potential drug-related effects. Since this is a single-dose, drug-drug interaction study, patients participating in this trial were not expected to gain any therapeutic benefit from co-administration of migalastat HCl with ERT. Although extended exposure to either dose of migalastat has yet to be performed in a co-administration setting, the safety and tolerability of multiple 150 mg QOD doses and multiple dose regimens to 500 mg per day have been well established in Fabry patients.

## Methods

Prior to patient enrollment, each investigator provided written Institutional Review Board or Ethics Committee approval to conduct the study at each study center, and at the time of enrollment, all study patients provided written informed consent before partaking in any study procedure: Western Institutional Review Board #20102043 (DGW, MH, OGA), Comite d' ethique de la recherche et de l' evaluation des technologies de la sante #2011–635 (DGB), Melbourne Health Human Research Ethics Committee 2012.019 (KN), Bellberry Human Research Ethics Committee #2012-03-722 (MT), Ethisch Comite Universitair Ziekenhuis Antwepen # 11/28/207 (FE), and Emory University Institutional Review Board #IRB00047460 (SS1).

The primary objectives of the study were to characterize the effects of 150 mg and 450 mg migalastat HCl orally administered 2 hours prior to initiation of agalsidase infusion on the safety and pharmacokinetics (PK) of active agalsidase, and to characterize the effect of agalsidase on the safety and PK of 150 mg of migalastat HCl in male patients with Fabry disease, most (18 of 23, 78.3%) of whom had non-amenable *GLA* mutations. A secondary objective was to characterize the effect of both doses of migalastat HCl on the tissue levels (skin) of active α-Gal A. An exploratory objective was to characterize the effect of both doses of migalastat HCl on peripheral blood mononuclear cells (PBMCs) the levels of active α-Gal A.

**Fig 1:**
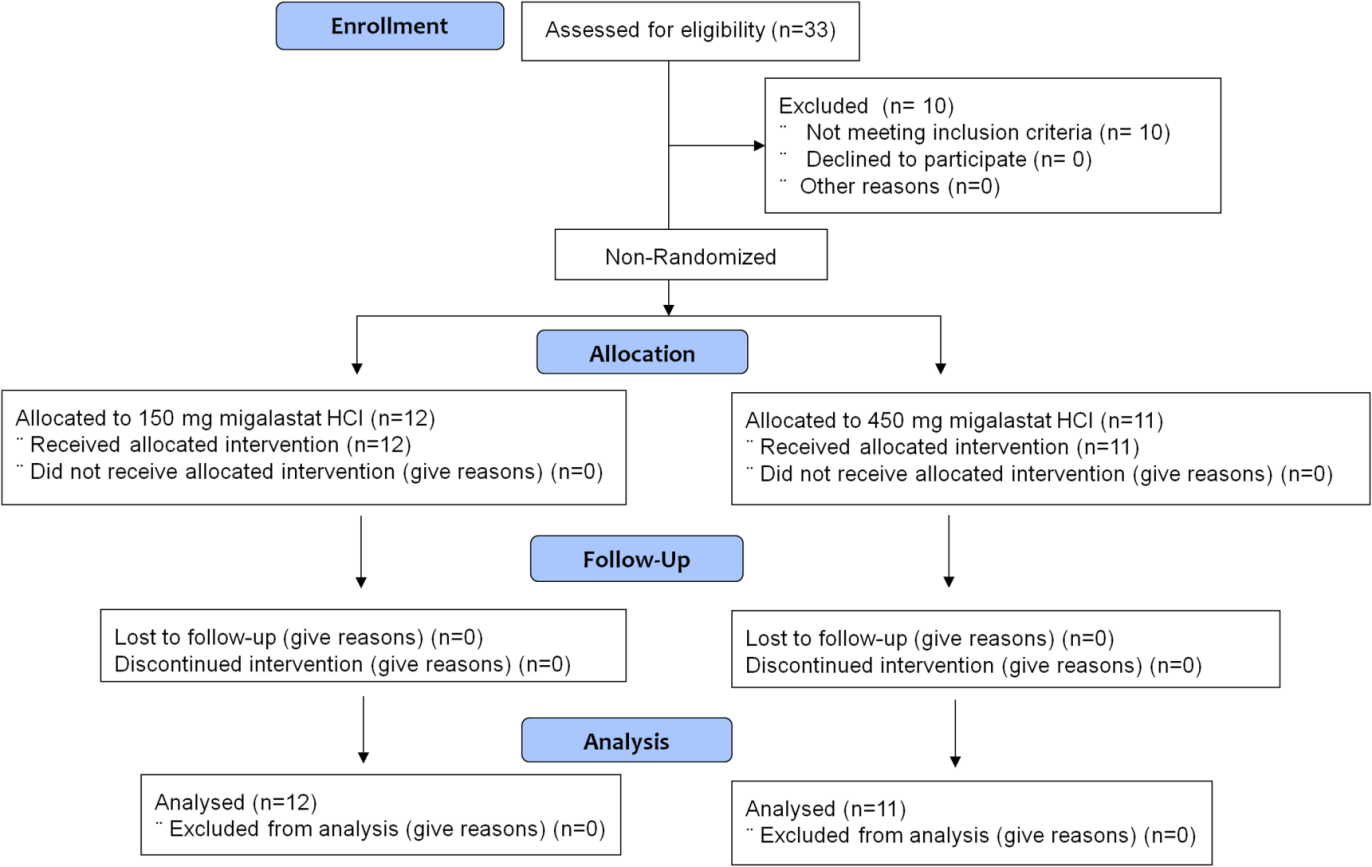
Flow diagram of subject disposition. Stage 1 was comprised of 3 periods. Period 1 patients received ERT alone on Day 1, Period 2 patients received ERT co-administered with 150 mg migalastat HCl on Day 1, and Period 3 patients received migalastat HCl alone on Day 7 (*i*.*e*., 7 days after the previous ERT infusion). A new cohort of patients was recruited for Stage 2. Stage 2 was comprised of 2 periods. Period 1 patients received ERT alone on Day 1 and Period 2 patients received ERT co-administered with 450 mg migalastat HCl on Day 1.

This was an open-label study comprised of two sequential, dose-ascending stages. In both stages patients underwent screening to determine eligibility followed by a multi-period, fixed-sequence design. Stage 1 was comprised of 3 periods. During Period 1, patients received their regularly scheduled infusion of agalsidase alone. Three doses of agalsidase were evaluated: the labeled dose of agalsidase alfa, 0.2 mg/kg, the labeled dose of agalsidase beta, 1.0 mg/kg, or the half-dose of agalsidase beta due to the temporary shortage, 0.5 mg/kg. Following a minimum 2-week dosing interval, the same patients returned for Period 2 and were co-administered 150 mg migalastat HCl 2 hours prior to initiation of infusion of agalsidase. To properly characterize the PK of α-Gal A activity, the dose and duration of agalsidase infusion were kept as close to identical as possible for Periods 1 and 2. During Period 3, patients received 150 mg migalastat alone on Day 7 (*i*.*e*., 7 days after their third ERT infusion). Stage 2 was performed in a new cohort of patients and was comprised of only 2 periods identical in design to Periods 1 and 2 of Stage 1, but with a 450 mg migalastat HCl dose administered 2 hours prior to initiation of agalsidase infusion during Period 2. Since there are no human safety data from co-administration of migalastat HCl and agalsidase, increasing the dose from 150 mg migalastat HCl to the 450 mg dose level with agalsidase was not made until thorough evaluation was completed of all available safety and tolerability information from at least the first 4 patients dosed with 150 mg migalastat HCl in conjunction with agalsidase. Skin biopsies were taken from various areas on the body (either arm, thigh, chest, buttock, or hip) using a 3 mm “punch” device on Day -1 (baseline) and at 24 hours (Day 2) and 144 hours (Day 7) of Periods 1 and 2. A flow diagram of subject disposition is provided in Fig 1.

All eligible study patients were male, diagnosed with Fabry disease, and between 18 and 65 years of age, inclusive; had a Body-Mass Index (BMI) between 18–35; initiated treatment with agalsidase at least 1 month, and had received at least two infusions, prior to screening; received a stable regimen and form (*i*.*e*., alfa or beta) of agalsidase (stable dose defined as not varying by more than ± 20%) for at least 1 month prior to screening; had an estimated creatinine clearance ≥ 50 mL/min (creatinine clearance was estimated using the 4-parameter Modification of Diet in Renal Disease (MDRD) equation: eGFR (mL/min/1.73 m^2^) = 186 x (Scr)^-1.154 ^x (Age)^-0.203 ^x (0.742 if female) x (1.212 if African-American); agreed to use medically accepted methods of contraception during the study and for 30 days after study completion. A patient was not considered for enrollment for any of the following documented clinical conditions: transient ischemic attack, ischemic stroke, unstable angina, or myocardial infarction within the 3 months prior to screening; clinically significant unstable cardiac disease (*e*.*g*., cardiac disease requiring active management, such as symptomatic arrhythmia, unstable angina, or NYHA class III or IV congestive heart failure); had a history of allergy or sensitivity to study drug (including excipients) or other iminosugars (e.g., miglustat, miglitol); required Glyset (miglitol) or Zavesca (miglustat) therapy; received investigational/experimental drug or device within 30 days prior to screening, except for use of investigational ERT; had an intercurrent illness or condition that may have precluded the patient from fulfilling the protocol requirements or suggested to the investigator that the potential patient may have had an unacceptable risk by participating in this study.

Primary outcome measures included plasma agalsidase PK parameter values (C_max_, t_max_, AUC, and t_½_) by measurement of α-Gal A activity after agalsidase infusion alone and when co-administered with migalastat HCl; plasma migalastat PK parameter values (C_max_, t_max_, AUC, and t_½_) following single oral administration alone and when co-administered with agalsidase; safety variables including adverse events, clinical laboratory tests, 12-Lead ECGs, physical examinations, vital signs, and infusion reactions. Tissue (skin) levels of active α-Gal A 24 hours and 7 days after administration of agalsidase alone or when co-administered with migalastat HCl (relative to baseline) were evaluated as secondary outcomes. Exploratory measurements included α-Gal A activity in PBMCs determined before initiation of the agalsidase infusion and 2, 4, and 24 hours, and 7 and 14 days, post-administration.

Plasma migalastat was analyzed using a validated liquid chromatography—tandem mass spectrometry (LC-MS/MS) method. The analytical range of the assay was 5.88 to 2940 ng/mL.

Determination of α-Gal A activity in human plasma, PBMC lysates, and skin homogenates was performed using 4-methylumbelliferyl-α-D-galactopyranoside (4-MUG) as the substrate. [17, 24] The product of the reaction was quantified by measuring against a 4-methyl-umbelliferone (4-MU) standard curve. Negative control sample was used as the background on all occasions.

Plasma enzymatic activity was calculated using the following equation:

$$Enzymatic\;Activity\;\left(\frac{\frac{nmol}{mL}}{hr}\right)=\frac{\left[4MU\;\left(\frac{pmol}{mL}\right)\times \frac{Dilution\;Factor}{1000}\right]}{Incubation\;Time\;(hr)}$$

Skin and PBMS lysate enzymatic activity were calculated using the following equation:

$$Enzymatic\;Activity\;\left(\frac{\frac{pmol}{mg}}{hr}\right)=\frac{\left[4MU\;\left(\frac{pmol}{mL}\right)\times Assay\;Vol\;(mL)\right]}{Protein\;C\;(\frac{mg}{mL})\times Protein\;V\;(mL)\times Incubation\;T\;(hr)}$$

The highest dilution factors within the linearity range of the standard curve were reported.

The safety and tolerability of migalastat were evaluated by monitoring clinical signs and symptoms, vital signs, and adverse events, and through the results of physical examinations, ECGs, and clinical laboratory tests (hematology, clinical chemistry, and urinalysis).

Serial blood sampling for plasma α-Gal A activity following agalsidase beta infusion was performed for 24 hours on Day 1 of Periods 1 and 2 for both Stages 1 and 2 pre-dose (Time 0) and at 0.5, 1, 1.5, 2, 2.5, 3, 4, 5, 6, 7, 8, 12, and 24 hours after initiation of infusion. Serial blood sampling for plasma α-Gal A activity following agalsidase alfa infusion was performed for 24 hours on Day 1 of Periods 1 and 2 for both Stages 1 and 2 pre-dose (Time 0) and at 0.33 (20 min), 0.66 (40 min), 1, 1.5, 2, 3, 4, 5, 6, 7, 8, 12, and 24 hours after initiation of infusion. Serial blood sampling for plasma migalastat concentrations following 150 mg or 450 mg migalastat HCl of Period 2 for both Stages 1 and 2 were performed pre-dose (Time 0), and at 1, 2, 3, 4, 5, 6, 7, 8, 10, 12, and 26 hours post-dose. Serial blood samples for plasma migalastat concentrations following 150 mg migalastat HCl of Period 3 for Stage 1 were performed pre-dose (Time 0), and at 1, 2, 3, 4, 5, 6, 7, 8, 10, 12, and 24 hours post-dose. Skin biopsies for α-Gal A activity were performed at baseline (pre-dose on Day 1 of Period 1 for Stages 1 and 2), and at 24 hrs (Day 2) and 144 hrs (Day 7) of Periods 1 and 2 for Stages 1 and 2. Blood samples for α-Gal A activity in PBMCs were taken at pre-dose, 2 and 4 hours on Day 1, 24 hours on Day 2, 144 hours on Day 7, and 336 hours on Day 14 after initiation of infusion of agalsidase for Periods 1 and 2 of both Stages 1 and 2.

Plasma α-Gal A activity and migalastat PK parameters (AUC_0-t_, AUC_0–∞_, C_max_, t_max_, k_el_, and t_½_) were determined by non-compartmental analysis using WinNonlin software (version 5.2 or higher). Pharmacokinetic parameters were summarized by treatment using descriptive statistics. Plasma α-Gal A and migalastat AUC_0-∞_ ratios for co-administration relative to agalsidase or migalastat HCl alone were calculated for each patient.

The impact of period/treatment in each stage on AUC_0-t_, AUC_0-∞_, and C_max_ of plasma migalastat and α-Gal A activity was assessed based on linear mixed effects model analyses. The point estimates for geometric means and their associated 90% confidence intervals (CIs) for the test–reference ratios were provided. Descriptive statistics were provided for PK data by treatment group. Descriptive statistics included N, arithmetic and geometric means, standard deviation, coefficient of variation, median, minimum and maximum values.

## Results

A total of 23 male Fabry patients were enrolled into the study, the first observation taking place on February 2, 2011, the last observation on October 9, 2012. Twelve patients were enrolled into Stage 1, five of whom received an IV infusion of a half-dose (0.5 mg/kg) of agalsidase beta alone during Period 1, and three of whom received the full, labeled dose (1.0 mg/kg). The same agalsidase beta dosages for each patient were co-administered with a single dose of 150 mg migalastat HCl during Period 2. A single dose of 150 mg migalastat alone was administered during Period 3. The half-dose of agalsidase beta was a direct result of a Fabrazyme shortage that occurred from March, 2011 through December, 2011. Four of the 12 Stage 1 patients received an IV infusion of 0.2 mg/kg labeled dose of agalsidase alfa alone during Period 1. This was followed by an IV infusion of 0.2 mg/kg agalsidase alfa co-administered with a single dose of 150 mg migalastat HCl during Period 2. A single oral dose of 150 mg migalastat alone was administered during Period 3.

Eleven male patients were enrolled into Stage 2 of the study. Seven of the 11 patients received agalsidase beta, six of whom received 1.0 mg/kg alone during Period 1, while the other received 0.5 mg/kg alone. The same agalsidase beta dosages for each patient were co-administered with a single dose of 450 mg migalastat HCl during Period 2. Four of the 11 Stage 2 patients received 0.2 mg/kg agalsidase alfa alone during Period 1, followed by co-administration with a single dose of 450 mg migalastat HCl during Period 2. All 23 patients completed the study. A summary of the disposition of patients is presented in Table 1.

**Table 1:** Patient Disposition.

Agalsidase Dose	Stage 1	Stage 2	Total
1.0 mg/kg agalsidase beta	3	6	9
0.5 mg/kg agalsidase beta	5	1	6
0.2 mg/kg agalsidase alfa	4	4	8
**Total**	**12**	**11**	**23**

There were no clinically meaningful differences between Stage 1 and 2 Fabry patients for age, sex, or body-mass index. Demographic characteristics are presented in Table 2. Generally, clinical characteristics of the Fabry population in this study were similar between migalastat dose groups.

**Table 2:** Demographic and Clinical Characteristics Related to Fabry Disease by Study Stage and Overall.

Characteristic	Stage 1	Stage 2	Total
	(N = 12)	(N = 11)	(N = 23)
No. (%) Sex	12 Males (100%)	11 Males (100%)	23 Males (100%)
No. (%) Race	12 White (100%)	11 White (100%)	23 White (100%)
No. (%) with Non-amenable Mutation^a^	11 (91.7%)	7 (63.6%)	18 (78.3%)
Mean (SD) Age (years)	47.0 (6.84)	38.9 (11.1)	43.7 (9.59)
Mean (SD) Body-Mass Index	23.9 (3.71)	23.5 (3.35)	23.7 (3.48)
Mean (SD) eGFR (mL/min)	74.6 (17.4)	86.9 (28.1)	80.8 (22.8)

^a^ See S1 Table for a complete listing of *GLA* mutations by patient.

**Fig 2:**
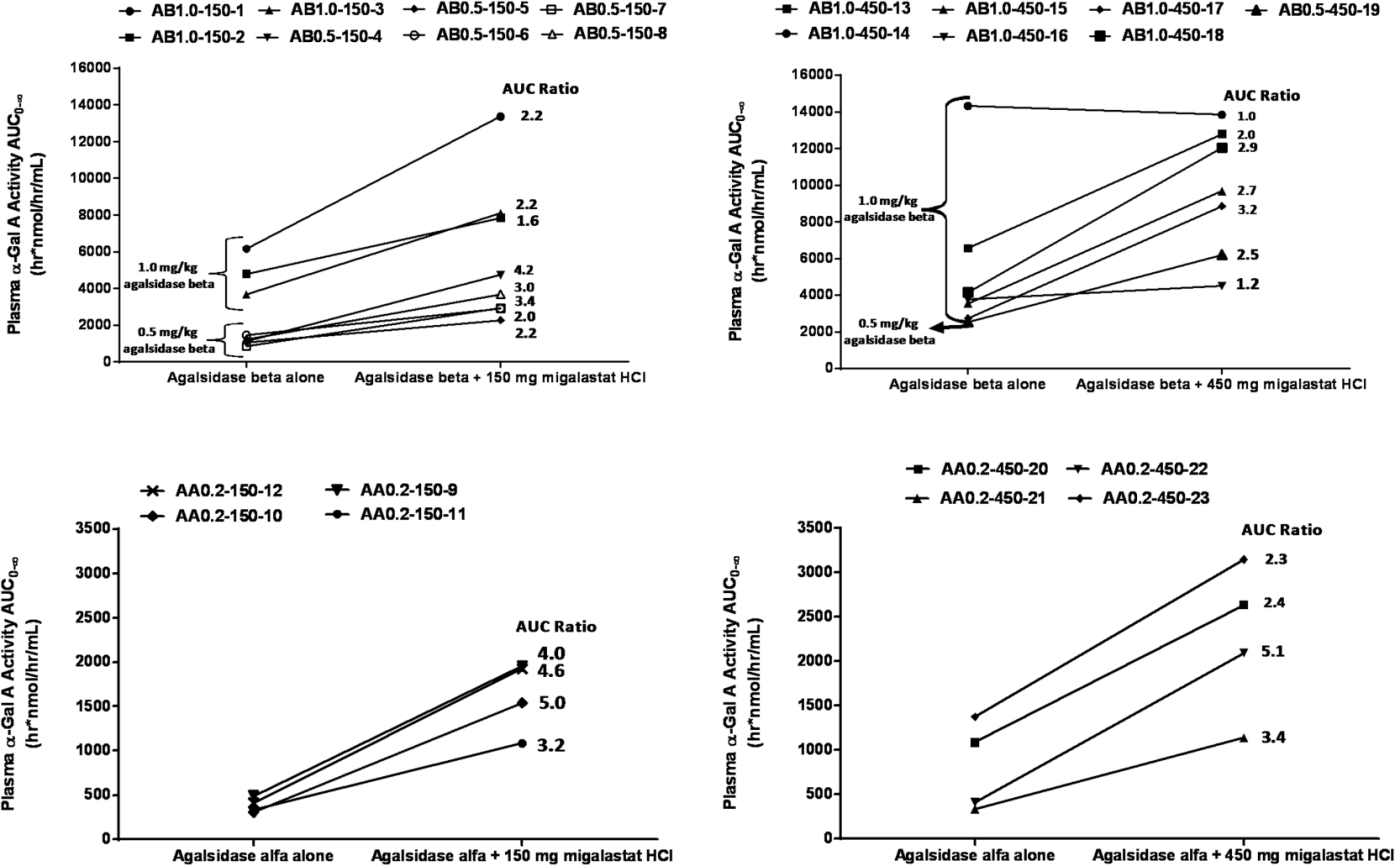
Two-by-two panel of total plasma α-Gal A activity AUC stick plots for agalsidase alone and co-administered with migalastat HCl. Upper left and right plots show individual patient changes between agalsidase beta alone and when co-administered with 150 mg migalastat HCl (upper left) or 450 mg migalastat (upper right) for plasma α-Gal activity AUC_0-∞_. Lower left and right plots show individual patient changes between agalsidase alfa alone and when co-administered with 150 mg migalastat HCl (lower left) or 450 mg migalastat HCl (lower right) for plasma α-Gal activity AUC_0-∞_. Patients’ actual ID number is blinded with the following code: Treatment and Dose (AB = agalsidase beta, 0.5 = 0.5 mg/kg dose or 1.0 = 1.0 mg/kg dose; AA = agalsidase alfa, 0.2 = 0.2 mg/kg dose)–migalastat HCl dose (150 = 150 mg, 450 = 450 mg)–arbitrary sequential number (1 through 23). Increases in plasma α-Gal activity AUC_0-∞_ are observed for all patients with the exception of AB1.0-450-14 shown in the upper left plot.

Of the 3 doses of agalsidase evaluated in this study, it should be noted that plasma α-Gal A activity exposures were non-linear with respect to dose. A two-fold increase in dose from 0.5 mg/kg to 1.0 mg/kg agalsidase beta alone resulted in an approximately 3.8-fold increase in α-Gal A activity AUC_0-∞_. A five-fold increase in dose from 0.2 mg/kg agalsidase alfa to 1.0 mg/kg agalsidase beta alone resulted in an approximately 9.7-fold increase in α-Gal A activity AUC_0-∞_ (S1 Fig). When evaluating agalsidase alone vs. co-administration with migalastat HCl, plasma AUC_0-∞_ of total α-Gal A enzyme activity increased for all 12 (100%) Stage 1 Fabry patients who were co-administered 150 mg migalastat HCl with agalsidase beta both at 0.5 mg/kg (2.0- to 4.1-fold, mean 2.8-fold), 1.0 mg/kg (1.6- to 2.2-fold, mean 2.0-fold), or agalsidase alfa at 0.2 mg/kg (3.2- to 5.0-fold, mean 4.2-fold) relative to agalsidase administered alone (Fig 2 and Table 3). For all 12 Fabry patients who were co-administered 150 mg migalastat with agalsidase relative to agalsidase alone, the point estimate for the AUC_0-∞_ α-Gal A activity geometric mean ratio and the 90% confidence intervals (CIs) was 2.94 (2.43, 3.56), thus demonstrating statistically significant increases. One patient, AB0.5-150-6, had an unbalanced agalsidase beta infusion (Period 2 infusion was 40 minutes longer than Period 1), that resulted in a slower rate of increase in α-Gal A activity during the infusion phase. Nevertheless, this patient demonstrated an overall 2.0-fold increase in total α-Gal A plasma activity AUC_0-∞_ as a result of an accumulating rate of incremental activity during the elimination phase.

**Table 3:** Summary of α-Gal A Activity Pharmacokinetic Parameters by Treatment.

Treatment	N	C_max_ ^a^ (nmol/hr/mL)	t_max_ ^b^ (hr)	AUC_0-∞_ ^a^ (hr*nmol/hr/mL)	AUC Ratio^a^	t_½_ ^c^ (hr)
0.2 mg/kg agalsidase alfa alone	4	300 (29.6)	0.67 (0.67–1.0)	381 (21.6)	4.2 (19.1)	4.5 (3.1)
0.2 mg/kg agalsidase alfa + 150 mg migalastat HCl		511 (15.0)	0.67 (0.67–1.0)	1584 (25.1)		4.3 (1.5)
0.2 mg/kg agalsidase alfa alone	4	358 (30.6)	0.67 (0.67–1.0)	672 (63.6)	3.1 (39.3)	5.2 (3.6)
0.2 mg/kg agalsidase alfa + 450 mg migalastat HCl		605 (22.9)	0.67 (0.67–1.0)	2109 (38.1)		5.3 (2.5)
0.5 mg/kg agalsidase beta alone	5	509 (17.0)	2.0 (2.0–2.3)	1129 (19.0)	2.8 (29.6)	3.9 (2.1)
0.5 mg/kg agalsidase beta + 150 mg migalastat HCl		877 (26.2)	2.0 (2.0–3.0)	3192 (27.6)		3.5 (1.3)
0.5 mg/kg agalsidase beta alone	1	684	3.0	2524	2.5	6.5
0.5 mg/kg agalsidase beta + 450 mg migalastat HCl		1351	3.0	6198		3.5
1.0 mg/kg agalsidase beta alone	3	1646 (24.3)	2.0 (1.5–3.0)	4765 (25.5)	2.0 (15.9)	5.3 (4.1)
1.0 mg/kg agalisdase beta + 150 mg migalastat HCl		2292 (38.7)	2.2 (2.0–3.0)	9464 (31.9)		4.3 (1.7)
1.0 mg/kg agalsidase beta alone	6	1655 (38.6)	2.3 (2.0–4.0)	4931 (74.3)	2.0 (43.4)	3.1 (1.9)
1.0 mg/kg agalsidase beta + 450 mg migalastat HCl		2316 (33.1)	2.3 (2.0–4.0)	9676 (33.1)		5.0 (1.5)

^**a**^ Geometric mean (CV%)

^b^ Median (range)

^c^ Arithmetic mean (SD)

The plasma AUC_0-∞_ of α-Gal A enzymatic activity increased for 10 of 11 (90.1%) Stage 2 Fabry patients who received co-administration of 450 mg migalastat HCl with agalsidase beta both at 0.5 mg/kg (2.5-fold) and 1.0 mg/kg (1.4- to 3.2-fold, mean 2.0-fold), or agalsidase alfa at 0.2 mg/kg (2.3- to 5.1-fold, mean 3.1-fold) relative to agalsidase administered alone (Fig 2 and Table 3). For all 11 Fabry patients who were co-administered 450 mg migalastat with agalsidase relative to agalsidase alone, the point estimate for the AUC_0-∞_ α-Gal A activity geometric mean ratio and the 90% confidence intervals (CIs) was 2.38 (1.84, 3.07), again demonstrating statistically significant increases. One patient, AB1.0-450-14, who received 1.0 mg/kg agalsidase beta alone had an α-Gal A activity AUC_0-∞_ that was approximately 3.4-fold greater than the mean AUC_0-∞_ of all other patients who received 1.0 mg/kg agalsidase beta alone. Due to this relatively greater response to agalsidase beta alone, patient AB1.0-450-14 was the only patient enrolled in the study who did not show an increase in α-Gal A activity AUC_0-∞_ (ratio, 1.0) following co-administration with migalastat HCl (Fig 2). Given the small sample sizes, variability was moderate to high for AUC_0-∞_ with CV% ranging from 19.0% to 74.3% (Table 3).

The C_max_ for total plasma α-Gal A activity following co-administration with migalastat HCl was also increased for 22 of 23 Fabry patients (95.7%) relative to agalsidase administered alone. Mean migalastat-mediated relative increases in C_max_ of approximately 40 to 42% were greater for the lower doses of agalsidase (0.5 mg/kg agalsidase beta and 0.2 mg/kg agalsidase alfa) than the higher dose of agalsidase (1.0 mg/kg agalsidase beta), which was approximately 28% regardless of migalastat HCl dose. Overall, relative increases in total plasma α-Gal A activity exposures (AUC and C_max_) were not dependent on migalastat HCl dose.

**Fig 3:**
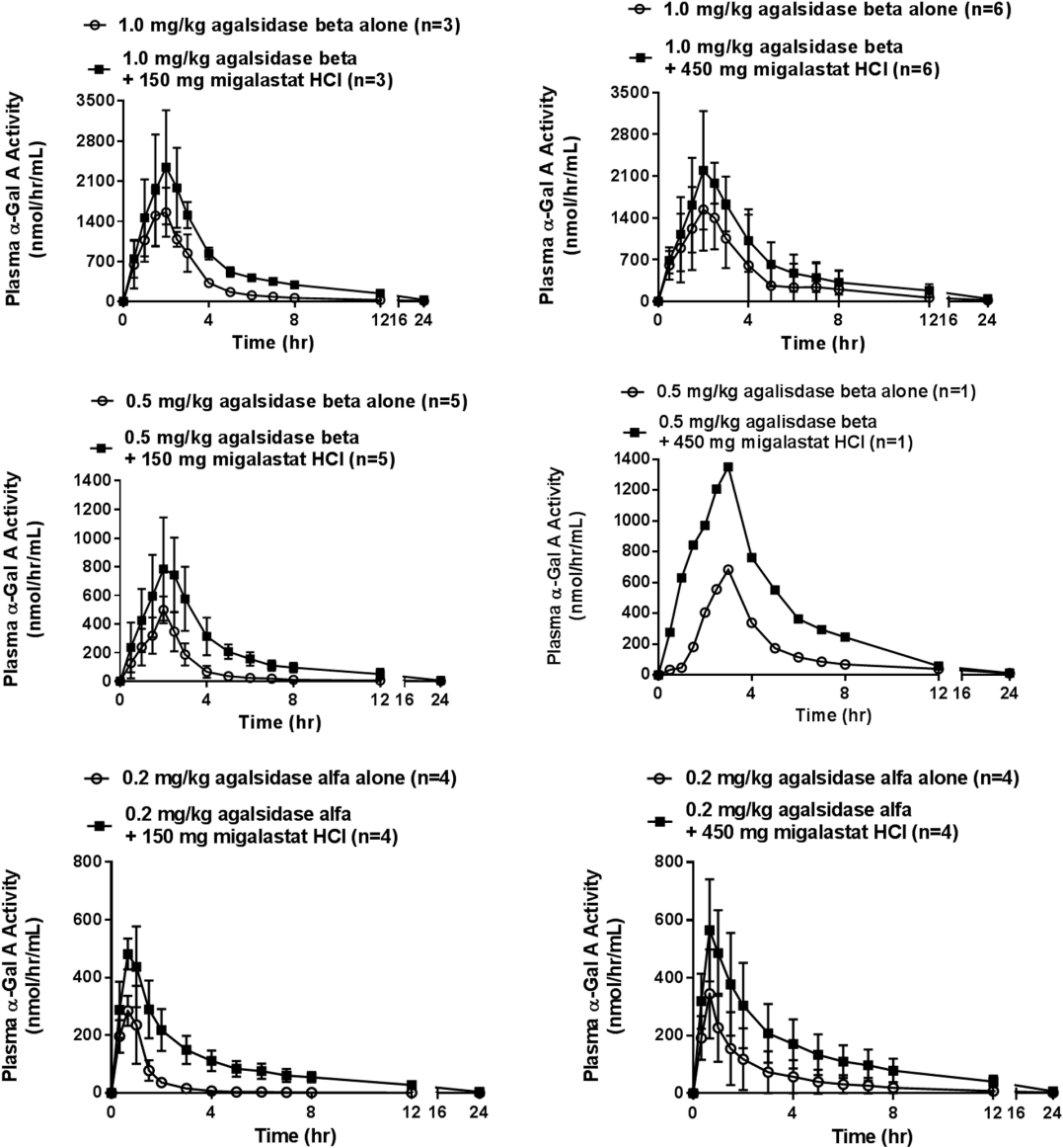
Mean (SD) total plasma α-Gal A activity-time profile panel for all treatments. Mean α-Gal A activity increased at all post-dose time points for all treatments after co-administration with migalastat HCl. Increases after co-administration do not appear to be related to migalastat dose.

Median t_max_ values for plasma α-Gal A activity were generally consistent with the duration of the infusion of agalsidase beta (two hours) and agalsidase alfa (40 minutes or 0.67 hours) (Table 3). Mean terminal half-life (t_½_) values were similar (approximately four hours) between co-administration of agalsidase with 150 mg migalastat HCl and agalsidase alone treatments (Table 4). Generally, increases in total plasma α-Gal A activity following co-administration started to occur during the infusion phase, peaked at the end of infusion, and continued during the terminal elimination phase at approximately the same rate of decline as agalsidase administered alone (Fig 3).

**Table 4:** Summary of α-Gal A Activity Levels in Skin.

	α-Gal A Activity in Skin (pmol/mg/hr)											
**ERT Treatment**	**Day 2 Absolute and Change from Baseline Mean (CV%)**											
	**Stage 1 (150 mg Migalastat HCl)**						**Stage 2 (450 mg Migalastat HCl)**					
	**n**	**BSL**	**Period 1 (ERT Alone)**	**Change from BSL**	**Period 2 (Co-ad)**	**Change from BSL**	**n**	**BSL**	**Period 1 (ERT Alone)**	**Change from BSL**	**Period 2 (Co-ad)**	**Change from BSL**
1.0 mg/kg Agalsidase Beta	3	472 (137)	1282 (51.3)	810 (12.6)	2680 (79.5)	2076 (61.4)	6	680 (43.6)	2616 (33.5)	1936 (43.1)	4699 (48.4)	4019 (55.9)
0.5 mg/kg Agalsidase Beta	5	509 (83.9)	843 (87.8)	334 (140)	2107 (104)	1509 (119)	1	119	208	89.4	766	647
0.2 mg/kg Agalsidase Alfa	4	147 (68.4)	316 (78.4)	121 (184)	535 (105)	388 (132)	4	69.8 (173)	262 (51.7)	169 (120)	519 (39.3)	412 (73.7)
	**Day 7 Absolute and Change from Baseline Mean (CV%)**											
1.0 mg/kg Agalsidase Beta	3	472 (137)	924 (102)	452 (65.5)	1187 (93.3)	715 (64.8)	6	680 (43.6)	1293 (64.5)	613 (112)	1469 (41.3)	789 (101)
0.5 mg/kg Agalsidase Beta	5	509 (83.9)	646 (71.7)	137 (61.4)	1083 (135)	574 (199)	1	119	190	71.6	354	235
0.2 mg/kg Agalsidase Alfa	4	147 (68.4)	270 (61.9)	136 (66.0)	360 (87.7)	214 (126)	4	69.8 (173)	224 (36.0)	182 (25.9)	349 (57.8)	293 (122)

BSL = Baseline

ERT = Enzyme replacement therapy

Co-ad = Co-administration of ERT with migalastat HCl

The ability of active α-Gal A to be taken up into cells and tissues was evaluated in this trial, as was the ability of migalastat HCl to affect these cellular and tissue levels. Five skin biopsy samples were taken from each Fabry patient: one at baseline (pre-dose, Period 1) and one on Days 2 (24-hour) and 7 post-administration of Periods 1 and 2. Twenty-one of 23 patients (91.3%) had evaluable matched skin biopsy samples for agalsidase alone on Days 2 and 7 vs. baseline. Similarly, twenty-one of 23 patients had evaluable matched skin biopsy samples for co-administration of agalsidase with migalastat HCl on Day 2, while 22 of 23 patients (95.7%) had evaluable matched skin biopsy samples for Day 7. The reasons for being unevaluable were insufficient sample quantity to measure α-Gal A activity, or the sample was misplaced at the clinic.

**Fig 4:**
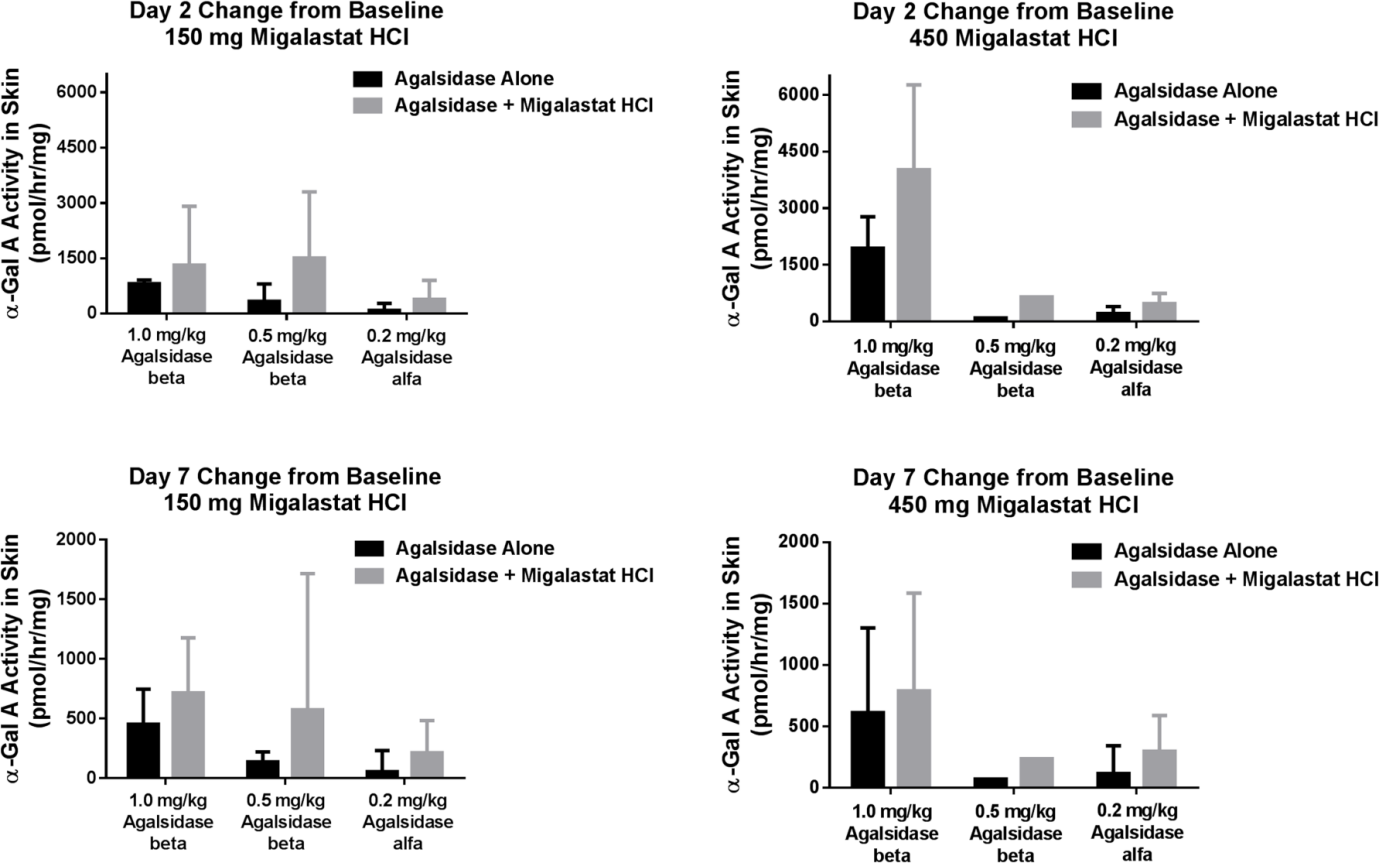
Mean (CV%) change from baseline on Days 2 and 7 for 1.0 mg/kg, 0.5 mg/kg, and 0.2 mg/kg agalsidase alone and agalsidase co-administered with 150 mg or 450 mg migalastat HCl.

Administration of agalsidase alone resulted in dose-related mean increases in α-Gal A activity in Day 2 and Day 7 skin biopsies. Co-administration of agalsidase with 150 or 450 mg migalastat HCl also resulted in agalsidase-dose-related increases in α-Gal A activity in Day 2 and Day 7 skin biopsies (Table 5, Fig 4). Importantly, the mean increases from baseline on Day 2 were approximately 2- to 3-fold greater when co-administered with migalastat HCl. Although mean increases from baseline appear to be migalastat dose-related, when evaluating α-Gal A ratios of co-administration with 150 mg migalastat to agalsidase alone vs. co-administration with 450 mg migalastat to agalsidase alone, mean increases appear similar (Fig 4). By Day 7, mean α-Gal A activity with or without migalastat, although still greater than baseline, had appreciably attenuated. Variability in skin α-Gal A activity, as expressed by CV%, was high (CV% > 50%) for most of the treatment groups. Formal statistical analyses on α-Gal A activity in skin were not performed.

**Table 5:** Plasma Migalastat PK Summary.

Treatment Group	C_max_ ^a^ (ng/mL)	t_max_ ^b^ (hr)	AUC_0-t_ ^a^ (ng∙hr/mL)	AUC_0-∞_ ^a^ (ng∙hr/mL)	AUC F_rel_ ^a^	t_½_ ^c^ (hr)
150 mg Migalastat HCl + Agalsidase (N = 12)	1626 (30.8)	3.0 (2–4)	13105 (39.4)	13521 (41.2)	-	5.1 (16.5)
150 mg Migalastat HCl Alone (N = 12)	1630 (30.0)	3.0 (2–4)	12371 (35.0)	12858 (36.2)	1.0 (33.7)	5.1 (17.2)
450 mg Migalastat HCl + Agalsidase (N = 11)	3935 (31.4)	4.0 (2–6)	31847 (31.5)	33101 (31.9)	2.5	4.9 (25.7)

^a^ Geometric mean (CV%)

^b^ Median (range)

^c^ Arithmetic mean (CV%)

Blood samples for isolation of PBMCs were taken at baseline (pre-dose, Period 1), and at 2 and 4 hours post-administration, and again on Days 2, 7, and 14 post-administration of Periods 1 and 2. The greatest increases in change from baseline for active α-Gal A in PBMCs were observed 2 hours post-administration for all treatment groups (S2 Table). When administered alone, dose-related increases of active α-Gal A activity were observed up to 24 hours post-administration. Co-administration with 150 mg migalastat HCl resulted in increases for change from baseline in active α-Gal A at all time points (S2 Table). When compared to agalsidase (beta or alfa) alone, sustained increases to 336 hrs (14 days) post-administration of active α-Gal A in PBMCs were observed following co-administration with 150 mg migalastat HCl. Although co-administration with 450 mg migalastat HCl also resulted in greater relative changes from baseline than agalsidase alone, the higher migalastat dose did not appear to have a greater effect on active α-Gal A levels in PBMCs (S2 Table). Formal statistical analyses on α-Gal A activity in PBMCs were not performed.

**Fig 5:**
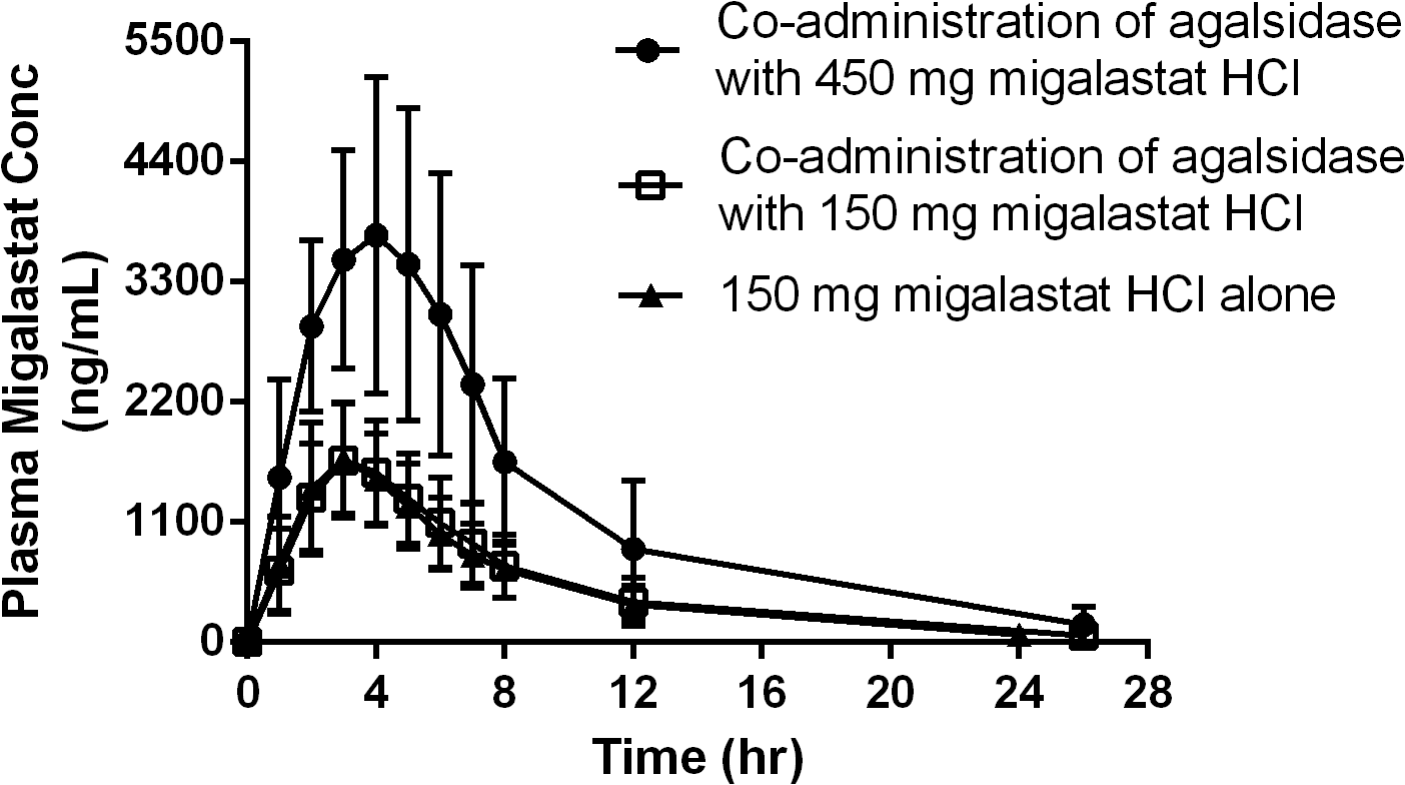
Mean (SD) plasma migalastat concentration-time profiles following co-administration of agalsidase with 450 mg or 150 mg migalastat HCl, and 150 mg migalastat HCl alone. Whether co-administered with agalsidase (Stage 1/Period 2) or administered alone (Stage 1/Period 3), plasma migalastat concentrations were similar at all post-dose time points. Mean concentration-time profiles following administration of 450 mg migalastat HCl (Stage 2/Period 2) were approximately dose proportional to 150 mg (Stage 1).

Mean (SD) plasma migalastat concentration-time profiles appear similar following co-administration of agalsidase (beta or alfa) with 150 mg migalastat HCl and following 150 mg migalastat HCl administered alone (Fig 5).

The 450 mg dose of migalastat HCl appears approximately dose proportional to the 150 mg dose for mean (SD) plasma migalastat concentration-time profiles and for both AUC_0-∞_ and C_max_ (Table 5). The time to maximum absorption of plasma migalastat (t_max_) was approximately three hours and the terminal elimination half-life was approximately five hours (Table 5).

Of the 23 Fabry patients enrolled in the study who received agalsidase alone or co-administered with migalastat HCl, 16 (69.6%) had at least one treatment-emergent adverse event (TEAE) during the study. No TEAE resulted in discontinuation from study treatment. Most (nine) of the TEAEs were mild in severity, five were considered moderate, and two were considered severe (acroparesthesia and monocular amaurosis fugax). Of these 16 patients, two had TEAEs that were considered study-drug related, and one patient had a TEAE that was serious. The study drug-related TEAEs were lethargy and nausea experienced by one patient after receiving 0.2 mg/kg agalsidase alfa alone, and bleeding post-skin biopsy experienced by one patient after receiving 0.2 mg/kg agalsidase alfa co-administered with 450 mg migalastat HCl. The incidents of lethargy and nausea were considered mild with probable/possible relationship to study medication (i.e., agalsidase) and resolved without sequalae. The bleeding was considered to be of probable relationship to the skin biopsy procedure (*i*.*e*., not specifically study-drug related), was mild in severity, and resolved. The serious TEAE was an episode of acute pain (acroparesthesia) related to Fabry disease (unrelated to study medication). The patient was hospitalized, treated with neurontin 300 mg t.i.d., and recovered without sequalae.

Changes in clinical laboratory evaluations, ECGs, vital signs, and physical examinations were unremarkable during the study.

## Discussion

In the current Phase 2 study, the ability to extend our preclinical proof-of-concept findings was explored for the first time in Fabry patients. The primary objective was to determine whether migalastat HCl co-administration with ERT is safe, and whether it can increase the exposure (AUC_0-∞_) of agalsidase (as measured by active α-Gal A levels in plasma), an indication of a positive drug-drug interaction.

Co-administration of migalastat HCl at doses of 150 mg and 450 mg with agalsidase alfa or beta was generally well-tolerated. No deaths or discontinuations of study treatment due to treatment-emergent adverse events were reported in this study. There were no clinically significant trends noted during the study in vital signs measurements, physical examinations, laboratory parameters, or ECG recordings. The evaluation of effect of plasma migalastat on agalsidase exposure was evaluated by comparing 24-hour exposures (AUC_0-∞_) of α-Gal A activity following co-administration with agalsidase and migalastat HCl relative to agalsidase administered alone. The PK evaluation was performed at two ascending, single-dose levels of migalastat HCl, 150 mg (Stage 1 of the study) followed by 450 mg (Stage 2 of the study). It should be noted that 5 of 8 of our agalsidase beta patients enrolled during Stage 1 were on a half-dose (0.5 mg/kg) due to a Fabrazyme shortage. By Stage 2 of the study, the shortage had ended, resulting in 6 of 7 of agalsidase beta patients having returned to the labeled dosage of 1.0 mg/kg. Both dose levels of migalastat HCl resulted in 2- to 4-fold increased plasma α-Gal A activity exposures following co-administration relative to agalsidase administered alone for 22 of 23 patients. One patient (AB1.0-450-14) showed no change in active α-Gal A plasma AUC_0-∞_ following co-administration with 450 mg migalastat HCl relative to 1.0 mg/kg agalsidase beta alone. It should be noted, however, that this patient had an approximately 3.4-fold greater active α-Gal A AUC following administration of 1.0 mg/kg agalsidase alone than the mean AUC_0-∞_ of all other patients who received 1.0 mg/kg agalsidase alone. Although the reason for this patient’s much greater response to 1.0 mg/kg agalsidase alone compared to all the others enrolled in this study has not been determined, there are several noteworthy facts regarding this patient’s migalastat PK profile. In this study, patients were to be orally administered migalastat HCl 2 hours prior to initiation of infusion of agalsidase so that t_max_ (3 hrs) would be reached approximately one hour into the infusion. Patient AB1.0-450-14 received 450 mg migalastat HCl at approximately 2.7 hours prior to initiation of infusion (approximate 45-minute delay of initiation of infusion). Additionally, this patient’s plasma migalastat exposure was 38.4% lower than the mean of all other patients who received 450 mg migalastat HCl in the study, t_max_ was reached in two hours rather than the median of 3 hours observed for most patients in the study, and plasma clearance was increased 43.5% relative to all other patients in the treatment group. These plasma migalastat PK factors, along with the exceptional response to agalsidase beta alone, could have contributed to the lack of response observed in this one patient. For all other patients, consistent with the preclinical findings, a 2- to 4-fold increase in α-Gal A activity was seen regardless of agalsidase or migalastat HCl dose. For instance, 150 mg migalastat HCl co-administration resulted in an average (n = 4) increase in plasma α-Gal A activity exposure of 4-fold with 0.2 mg/kg agalsidase alfa, 3-fold (n = 5) with 0.5 mg/kg agalsidase beta, and 2-fold (n = 6) with 1.0 mg/kg agalsidase. The 450 mg migalastat HCl co-administration resulted in an average (n = 4) increase in plasma α-Gal A activity exposure of 3-fold with 0.2 mg/kg agalsidase alfa, 2.5-fold (n = 1) with 0.5 mg/kg agalsidase beta, and 2-fold (n = 3) with 1.0 mg/kg agalsidase. Interestingly, the co-administration of either 150 or 450 mg migalastat HCl with 0.2 mg/kg agalsidase alfa increased the C_max_ of the enzyme to levels comparable to those achieved with 0.5 mg/kg agalsidase beta alone. Likewise, the co-administration of 150 mg or 450 mg migalastat HCl with 0.5 mg/kg agalsidase beta increased the C_max_ to levels comparable to those achieved with 1.0 mg/kg of agalsidase beta alone. These data indicate that migalastat HCl co-administration can increase the exposure of active agalsidase in the circulation.

Another primary objective of the study was to determine whether agalsidase affected plasma migalastat exposures. This was accomplished by calculating plasma migalastat AUC_0-∞_ ratios for co-administration to migalastat HCl administered alone and testing with the ANOVA. It was not anticipated that agalsidase would affect plasma migalastat exposure, which appeared to be the case as the ratio of geometric mean AUC_0-∞_ (CIs) was 1.05 (0.81, 1.37). Upon close examination of the data and statistical analysis, the percent differences between exposures for co-administration and migalastat HCl alone were high for some patients with resultant CV% of mean 41.2% for the co-administration treatment and 36.2% for the migalastat HCl alone treatment. A probable cause may be related to timing of meals around dosing. Migalastat HCl has a significant food effect, not only for a high fat meal, but for a light meal as well. Decreases in AUC_0-∞_ of approximately 40% have been observed in food effect studies when high fat or light meals were administered one hour before or after administration of migalastat HCl. [25] However, since no increasing or decreasing trend was observed for migalastat exposures when co-administered with agalsidase or taken alone, an AUC_0-∞_ ratio of approximately 1.0 provides satisfactory evidence that agalsidase did not affect migalastat exposures.

The secondary objectives of this study were to characterize the effect of co-administration of migalastat HCl on the distribution (uptake) of agalsidase to skin. Most (18 of 21, 85.7%) patients with evaluable biopsies demonstrated greater levels of active α-Gal A in skin on Day 2 following co-administration with either 150 or 450 mg migalastat HCl relative to agalsidase administration alone. Here again, due to the small sample sizes, statistically significant differences between cohorts could not be achieved; however, the overall migalastat-mediated increases of active α-Gal A in skin are quite consistent given the variability introduced by differing tissue sample weights, protein content, sites of biopsy, heterogeneity of the tissue, and dilution factors for homogenate preparation. The greatest increases from baseline in α-Gal A activity in skin were agalsidase dose-related, with the highest increases from baseline following 1.0 mg/kg agalsidase with 150 or 450 mg migalastat HCl, and the lowest following 0.2 mg/kg agalsidase alone. An average relative increase of up to 2.0-fold ([migalastat + agalsidase] / agalsidase alone) in active α-Gal A skin levels was seen in the majority of patients administered either dose of migalastat HCl (150 or 450 mg) in combination with agalsidase (0.2 mg/kg agalsidase alfa, or 0.5 or 1.0 mg/kg agalsidase beta).

Lastly, an exploratory objective of the study was to characterize the effect of co-administration of migalastat HCl on the distribution (uptake) of agalsidase into PBMCs. Although PBMCs are not a known target cell type of Fabry disease pathology, they do provide a minimally invasive and readily available cell source for evaluation of active α-Gal A uptake. Following co-administration with migalastat HCl, most patients (22 of 23, 95.7%) demonstrated greater increases from baseline in PBMC α-Gal A activity relative to agalsidase alone at all post-administration time points. One patient, AB1.0-450-18, had a 31.7-fold greater α-Gal A activity baseline in PBMCs than the mean of all other patients in the 1.0 mg/kg agalsidase with 450 mg migalastat HCl treatment group. This unusually high baseline activity level resulted in decreased changes from baseline from 24 hours to 336 hours post-administration for both treatments. However, the PBMC α-Gal A activity levels following co-administration were greater than agalsidase alone at all time points. Overall, the average relative increases in α-Gal A activity in PBMCs with migalastat HCl co-administration on agalsidase beta 24 hours, 7 days, and 14 days post-administration were 2- to 2.4-fold, 2- to 4.5-fold, and 2- to 3.2-fold, respectively. The only exception was patient AB0.5-450-19, the only patient enrolled in the 0.5 mg/kg agalsidase beta with 450 mg migalastat HCl cohort, who showed a 2.7-fold increase at 24 hours and a 5.5-fold increase at Day 7. On average, the relative increases in α-Gal A activity in PBMCs with migalastat HCl co-administration were 2- and 1.35-fold on Days 7 and 14, respectively.

The observed increases in α-Gal A activity levels in both plasma and tissues of this study provided proof-of-concept for co-administration of a PC with ERT in humans, and confirmed preclinical findings. [26] The single administration study showed increases in plasma α-Gal A activity exposures, suggesting increased stability and greater properly folded protein available for uptake into tissues. However, the α-Gal A activity increases did not appear to be migalastat HCl dose-related, at least in this single-dose study. As stated above, the number of patients enrolled in each cohort was relatively small, limiting the ability to evaluate differences between doses of migalastat HCl. Therefore, multiple-dose studies geared mainly towards clinical benefits such as substrate reduction and improvements of clinically relevant biomarkers with larger numbers of patients enrolled in each cohort are warranted. Overall, it can be concluded that the pharmacological consequences for both doses of migalastat HCl were improved exposure of active α-Gal A in plasma and greater levels of active enzyme in the cells and tissues that were evaluated in this study.

## Supporting Information

S1 Checklist Trend Statement Checklist. (PDF)

S1 Fig Mean (SD) total plasma α-Gal A activity-time profiles for agalsidase beta and alfa alone. Greater-than-dose-proportional increases are observed for agalsidase at doses of 0.2, 0.5, and 1.0 mg/kg. Agalsidase beta was infused for approximately 2 hours, as shown by peak concentrations for the 0.5 and 1.0 mg/kg doses. Agalsidase alfa was infused for approximately 45 minutes, as shown by the peak concentration at the 0.2 mg/kg dose.
(TIF)

S1 Protocol Final Protocol and Amendment. (PDF)

S1 Table *GLA* Genotypes. S1 Table includes the *GLA* genotype for each Fabry patient enrolled in the study. Most (18 of 23) Fabry patients had non-amenable mutations.
(DOCX)

S2 Table Summary of Total α-Gal A Activity Levels in PBMCs. S2 Table provides mean (CV%) changes from baseline in α-Gal A activity in PBMCs for ERT administered alone and when co-administered with migalastat HCl. Generally, the largest changes from baseline occurred from 2 to 4 hours post-dose during co-administration with migalastat HCl.
(DOCX)
