# Supplementary material for: Oral Migalastat HCl Leads to Greater Systemic Exposure and Tissue Levels of Active α-Galactosidase A in Fabry Patients when Co-Administered with Infused Agalsidase
Source: PLoS One. 2015 Aug 7;10(8):e0134341. doi: 10.1371/journal.pone.0134341 (PMC4529213; doi:10.1371/journal.pone.0134341)
Supplement: S1 Protocol — (PDF) [file pone.0134341.s003.pdf]

CLINICAL STUDY PROTOCOL

AN OPEN-LABEL PHASE 2A STUDY TO INVESTIGATE DRUG-DRUG INTERACTIONS BETWEEN AT1001 (MIGALASTAT HYDROCHLORIDE) AND AGALSIDASE IN SUBJECTS WITH FABRY DISEASE

Protocol No.: AT1001-013

ORIGINAL PROTOCOL: JULY 26, 2010

AMENDMENT 4: NOVEMBER 2, 2011

Compound: AT1001 (migalastat hydrochloride)

Sponsor

Amicus Therapeutics, Inc.
6 Cedar Brook Drive
Cranbury, NJ 08512
Phone: 609-662-2000

US IND Number: 68,456
EudraCT Number: 2010-022709-16

1. DECLARATIONS OF SPONSOR AND INVESTIGATOR

1.1. Declaration of Sponsor

This clinical study protocol was subject to critical review and has been approved by the sponsor. The information it contains is consistent with:

> The current risk-benefit evaluation of AT1001;
>
> The moral, ethical, and scientific principles governing clinical research as set out in the Declaration of Helsinki and the principles of Good Clinical Practice (GCP) as described in the US Code of Federal Regulations (CFR), Parts 50, 54, 56, and 312; the International Conference on Harmonisation (ICH) Guidelines for Good Clinical Practice (E6); and in the applicable local requirements.

The investigator will be supplied with details of any significant or new findings, including adverse events (AEs), relating to treatment with AT1001.

Date: 02/NOV/2011 Signature: [signature]

Mathews Adera, MD
Medical Director, Clinical Research
Amicus Therapeutics, Inc.

1.2. Declaration of Investigator

I confirm that I have read the clinical study protocol. I understand it, and I will work according to the principles of GCP as described in 21 CFR parts 50, 54, 56, and 312; the ICH Guidelines for Good Clinical Practice (E6); and according to applicable local requirements.

Investigator

Date: __________ Signature: ____________________________________

Printed Name: ________________________________

Institution: __________________________________

TABLE OF CONTENTS

1. DECLARATIONS OF SPONSOR AND INVESTIGATOR 2
1.1. Declaration of Sponsor 2
1.2. Declaration of Investigator 2
2. PROTOCOL OUTLINE 8
3. INTRODUCTION AND STUDY RATIONALE 28
3.1. Fabry Disease 28
3.2. AT1001 29
3.2.1. Nonclinical Summary 29
3.2.2. Clinical Summary 29
3.3. Agalsidase 30
3.3.1. Agalsidase alfa 30
3.3.2. Agalsidase beta 31
3.4. Effect of AT1001 on the Pharmacokinetics and Pharmacodynamics of α-Gal A 31
3.4.1. Effect of AT1001 on Fabrazyme® (agalsidase beta) 31
3.4.2. Effect of AT1001 on Replagal™ (agalsidase alfa) 32
3.5. Preliminary Safety Evaluation of Co-Administered AT1001 and Fabrazyme® in *GLA* Deficient Mice 32
3.6. Study Rationale 33
3.6.1. Clinical Rationale 33
3.6.2. AT1001 Dose Rationale 33
4. STUDY OBJECTIVES 35
4.1.1. Primary Objectives 35
4.1.2. Secondary Objective 35
4.2. Study Endpoints 35
4.2.1. Primary Endpoints 35
4.2.2. Secondary Endpoint 35
4.2.3. Exploratory Endpoints 35
5. STUDY DESIGN 36
5.1. Randomization and Blinding 36
6. SELECTION OF SUBJECTS 37
6.1. Target Population 37

6.2. Number of Subjects 37
6.3. Recruitment Strategy 37
6.4. Inclusion Criteria 37
6.5. Exclusion Criteria 37
7. STUDY TREATMENT 39
7.1. Details of Study Treatments 39
7.2. Dosage Schedule 39
7.3. Dose Escalation 39
7.4. Packaging and Labeling 40
7.5. Supplies and Accountability 40
7.6. Compliance 40
8. PRIOR AND CONCOMITANT ILLNESSES AND TREATMENTS 41
8.1. Prior and Concomitant Illnesses 41
8.2. Prior and Concomitant Treatments 41
8.3. Description of Study Visits 41
8.3.1. Screening 41
8.3.2. Stage 1 42
8.3.2.1. Period 1 42
8.3.2.2. Period 2 43
8.3.2.3. Period 3 44
8.3.3. Stage 2 45
8.3.3.1. Period 1 45
8.3.3.2. Period 2 46
8.3.4. Follow-Up Visit 48
8.3.5. Unscheduled Visits 48
8.4. Assessments 48
8.4.1. AT1001 and rhα-Gal A Pharmacokinetics 48
8.4.2. α-Gal A Enzyme Levels in Skin 48
8.4.3. WBC α-Gal A Levels 49
8.4.4. Plasma Lyso-GB3 49
8.4.5. Urine GL-3 and Lyso-GB3 49
8.4.6. Antibody Titer 49
8.4.7. Sample Handling and Shipment 49

8.4.8. Safety Parameters 50
8.4.9. Vital Signs, Weight and Height 50
8.4.10. ECG Monitoring 50
8.4.11. Clinical Laboratory Tests 50
8.4.12. Adverse Events 51
8.5. General and Dietary Restrictions 51
9. ADVERSE EVENTS 52
9.1. Definitions 52
9.1.1. Adverse Event 52
9.1.2. Serious Adverse Event 52
9.1.3. Relationship to Study Medication 52
9.1.4. Assessment of Severity 53
9.2. Reporting of Adverse Events 53
9.3. Additional Reporting Requirements for Serious Adverse Events 54
9.4. Reporting of Serious Adverse Reactions 55
9.5. Other Reporting Requirements 55
9.5.1. Reporting of Pregnancy 55
9.5.2. Reporting of Overdose 55
9.5.3. Reporting of Possible Study Medication Product Quality Defects 56
10. WITHDRAWALS 57
10.1. Withdrawal of Subjects 57
10.2. Replacement of Subjects 57
11. EMERGENCY PROCEDURES 58
11.1. Emergency Sponsor Contact 58
11.2. Emergency Treatment 58
12. DATA ANALYSIS 59
12.1. Analysis Populations 59
12.2. Primary Endpoints 59
12.2.1. Pharmacokinetic Parameters 59
12.2.1.1. Statistical Procedures 59
12.2.1.2. Statistical Analysis 59
12.2.2. Safety Evaluation (Primary Endpoint) 60
12.3. Secondary Endpoint 60

12.3.1. Skin α-Gal A Levels 60
12.4. Interim Analysis 60
12.5. Sample Size Calculation 60
13. ETHICAL AND LEGAL ASPECTS 61
13.1. Good Clinical Practice 61
13.2. Delegation of Investigator Duties 61
13.3. Subject Information and Informed Consent 61
13.4. Confidentiality 62
13.5. Protocol Amendments 62
13.6. Approval of the Clinical Study Protocol and Amendments 62
13.7. Ongoing Information for IEC/IRB 63
13.8. Closure of the Study 63
13.9. Record Retention 63
13.10. Liability and Insurance 64
13.11. Financial Disclosure 64
13.12. Protocol Adherence 65
14. STUDY MONITORING AND AUDITING 66
14.1. Study Monitoring and Source Data Verification 66
14.2. On-site Audits 67
15. DOCUMENTATION AND USE OF STUDY FINDINGS 68
15.1. Documentation of Study Findings 68
15.2. Use of Study Findings 68
16. REFERENCES 70

LIST OF TABLES

Table 1: Schedule of Assessments (Stages 1 and 2) 18
Table 2: FABRAZYME® (agalsidase beta) – Period 1, Stage 1 or 2 Blood Collection Times 20
Table 3: FABRAZYME® (agalsidase beta) – Period 2, Stage 1 or 2 Blood Collection Times 21
Table 4: FABRAZYME® (agalsidase beta) – Period 3, Stage 1 ONLY Blood Collection Times 22
Table 5: REPLAGAL™ (agalsidase alfa) – Period 1, Stage 1 or 2 Blood Collection Times 23
Table 6: REPLAGAL™ (agalsidase alfa) – Period 2, Stage 1 or 2 Blood Collection Times 24
Table 7: REPLAGAL™ (agalsidase alfa) – Period 3, Stage 1 ONLY Blood Collection Times 25
Table 8: Pharmacokinetic Sampling Time Windows 25
Table 9: Treatment Schedule 39

LIST OF FIGURES

Figure 1: Study Schematic 17

2. PROTOCOL OUTLINE

**Name of Sponsor/Company:** Amicus Therapeutics
**Name of Investigational Product:** AT1001
**Name of Active Ingredient:** Migalastat hydrochloride
**Title of Study:** A Phase 2a Study to Investigate Drug-Drug Interactions Between AT1001 (migalastat hydrochloride) and Agalsidase in Subjects with Fabry disease
**Protocol Number**: AT1001-013
**Study Sites**: Approximately 5
**Development Phase**: Phase 2a
**Study Duration:** Up to 4.5 months

Objectives

This study will provide drug-drug interaction information after co-administration of AT1001 and agalsidase. In addition, information on the effect of 150 mg and 450 mg doses of AT1001 on agalsidase will be obtained for proof of concept that AT1001 has the potential to improve the pharmacokinetic properties of agalsidase. Patients receiving either agalsidase alfa (Replagal™) or agalsidase beta (Fabrazyme®) will be eligible to participate in this study. The results of this study may support further development of AT1001 in combination with Enzyme Replacement Therapy (ERT).

The primary objectives of this study are:

- To characterize the effects of 150 mg and 450 mg of AT1001 administered 2 hours before administration of agalsidase on the safety and plasma pharmacokinetics of agalsidase in subjects with Fabry Disease
- To characterize the effect of agalsidase on the safety and plasma pharmacokinetics of 150 mg of AT1001 administered 2 hours before administration of agalsidase in subjects with Fabry Disease

The secondary objective of this study is:

- To characterize the effect of 150 mg and 450 mg AT1001 on the distribution of α-Gal A to skin after administration of agalsidase

Primary Endpoints:

- AT1001 plasma pharmacokinetic parameter values after administration of a single oral dose of AT1001 alone and in combination with agalsidase
- Agalsidase plasma pharmacokinetic parameter values by measurement of α-Gal A enzyme levels and protein levels after agalsidase infusion alone and in combination with AT1001
- Safety variables: adverse events, clinical laboratory tests, 12-Lead ECGs, physical examinations, vital signs, and infusion reactions

Secondary Endpoint:

- Distribution of agalsidase to skin after dosing with agalsidase alone and agalsidase in combination with AT1001 at 24 hours and 7 days after dosing by measuring α-Gal A levels and protein levels

Exploratory Endpoints:

- Urinary GL-3 excretion before and 14 days after each agalsidase dose
- GL-3 in skin after dosing with agalsidase alone and agalsidase in combination with AT1001 at 24 hours and 7 days after dosing
- WBC α-Gal A enzyme levels, determined before initiation of the agalsidase infusion and at 2, 4, and 24 hours and 7 and 14 days after dosing
- Antibody titer (Immunoglobulin G (IgG)) before initiation of an infusion of agalsidase
- Plasma lyso-GB3 concentrations and urinary excretion of lyso-GB3 before each dose of agalsidase and 14 days after each dose of agalsidase

All plasma, WBC and skin measurements of α-Gal A enzyme levels will be performed with and without concanavalin A (Con A) capture and determination of protein levels will be by Western blot.

Study Design

This open-label study will consist of two stages (see Figure 1). Stage 1 will consist of screening and a three-period study to evaluate the effect of 150 mg AT1001 on the pharmacokinetics and safety of agalsidase and the effect of agalsidase on the pharmacokinetics and safety of 150 mg AT1001. Stage 2 will consist of screening and a two-period study to evaluate the effect of 450 mg AT1001 on the pharmacokinetics and safety of agalsidase. In Stage 2, the effect of agalsidase on the pharmacokinetics and safety of a 450 mg dose of AT1001 will not be evaluated; the plasma exposure of AT1001 will be characterized when AT1001 is administered with agalsidase solely to confirm the attainment of adequate AT1001 plasma concentrations.

Male subjects between 18 and 65 years of age who have been receiving a stable dose (0.3-1.0 mg/kg) of agalsidase beta or (≥ 0.2 mg/kg) of agalsidase alfa for at least one month before study entry and who meet all other eligibility criteria will be enrolled into the study. A stable dose of enzyme is defined as a dose not varying by more than ±20%. The first ten to twelve subjects (a minimum of 4 subjects receiving agalsidase alfa; the remaining subjects receiving agalsidase beta) meeting all eligibility criteria will be enrolled into Stage 1. The decision to initiate dosing in Stage 2 will be made by the Amicus Medical Monitor and Investigator(s) after consideration of safety and tolerability information from at least the first 4 subjects having completed Stage 1. Subjects on agalsidase beta will be assigned to Stage 2 after a minimum of six subjects complete Stage 1. Subjects on agalsidase alfa will be assigned to Stage 2 after a minimum of four subjects complete Stage 1. At least four subjects will be treated with 450 mg AT1001 co-administered with each form of ERT in Stage 2.

Subjects may repeat a treatment period under certain exceptional circumstances, such as changes in their infusion rates or durations, which make their data non-evaluable. Consultation with the Amicus medical monitor is required prior to any such re-challenge.

Since this is a single-dose, drug-drug interaction study, subjects participating in this trial are not expected to gain any therapeutic benefit.

Number of Subjects

The total number of subjects will be approximately 18 to 24 evaluable subjects. Non-evaluable subjects may be replaced. Subjects who enroll in Stage 1 may be re-enrolled for Stage 2 if they provide written consent and agree to all study procedures. A washout period of at least 30 days post-migalastat HCl will be required prior to the first ERT dosing in Stage 2.

Study Procedures

Screening

Informed consent will be obtained before any study-specific procedures are performed. The screening visit must occur within 28 days of Period 1 (Day -1) to determine eligibility. Assessments and procedures to be performed at the screening visit will include: review entry criteria, medical history (including *GLA* genotype, if known), height, weight, concomitant/prior medication assessment, physical exam, vital signs, 12-Lead ECGs, clinical laboratory tests (hematology, serum chemistry, coagulation profile, and urinalysis), and estimated glomerular filtration rate (eGFR).

Stage 1

Each subject will receive each of the following treatments in the order described below:

Period 1: Agalsidase alone as an intravenous (IV) infusion

Period 2: A 150 mg oral dose of AT1001 two hours before initiation of an intravenous infusion of agalsidase

Period 3: A 150 mg oral dose of AT1001

Note: the dose and infusion length of agalsidase administered in Periods 1 and 2 must be identical. Agalsidase alfa will be administered as a 40-minute intravenous infusion; agalsidase beta will be administered as a 2-hour intravenous infusion.

Period 1

Subjects meeting all eligibility criteria will be admitted to the clinical site approximately 10 hours prior to their next scheduled agalsidase infusion (Day -1). Subjects will have the following assessments performed at check-in: adverse event assessment, concomitant medications, physical exam, weight, vital signs, 12-lead ECG, clinical laboratory tests (serum chemistry, hematology, and urinalysis), urine GL-3/lyso GB-3, and skin biopsy (punch biopsy for measurement of α-Gal A enzyme levels; if sufficient sample is available, skin GL-3 will also be determined).

On the morning of Day 1, urine (first morning void) will be collected for urinary GL-3 and lyso-GB3 determinations followed by administration of the subject’s current agalsidase dose given as an infusion using an infusion pump.

Blood samples for pharmacokinetic and pharmacodynamic analysis will be collected immediately before initiation of the agalsidase infusion and over a 24-hour period after initiation of the agalsidase infusion. Plasma and WBC α-Gal A enzyme levels, plasma lyso-GB3 and plasma antibody titer will be determined from the collected blood samples at the times summarized in Table 2 for agalsidase beta and in Table 5 for agalsidase alfa. A 12-lead

ECG will be performed at the end of the agalsidase infusion, immediately after collection of the post-infusion blood sample. In addition, vital signs will be collected and adverse events and concomitant medications will be assessed.

On Day 2, a punch skin biopsy will be collected 24 hours after initiation of the previous day's infusion from which α-Gal A enzyme levels will be determined; if sufficient sample is available, skin GL-3 will also be determined. After collection of the last pharmacokinetic sample and before discharge, the following assessments will be performed: adverse event assessment, concomitant medications, physical exam, weight, vital signs, and clinical laboratory tests (serum chemistry, hematology, a blood sample for plasma and WBC α-Gal A, and urinalysis).

On Day 7, subjects will return to the clinical site and have the following assessments performed: physical exam, vital signs, concomitant medications, and adverse event assessment. A skin biopsy will be collected from which α-Gal A enzyme levels will be determined; if sufficient sample is available, skin GL-3 will also be determined. A blood sample for WBC α-Gal A, plasma lyso GB-3, and plasma enzyme level determinations will also be collected.

On Day 14, a urine sample (first morning void) for determination of urinary GL-3 and lyso-GB3 excretion will be collected. A blood sample for WBC α-Gal A, plasma lyso GB-3, and plasma enzyme level determinations will also be collected and vital signs, adverse events, and concomitant medications will be assessed.

Period 2

Subjects will be re-admitted to the clinical site approximately 10 hours prior to their next scheduled agalsidase infusion (Day -1). Subjects will have the following assessments performed at check-in: adverse event assessment, concomitant medications, physical exam, weight, vital signs, 12-lead ECG, clinical laboratory tests (serum chemistry, hematology, and urinalysis), and urine GL-3/lyso GB-3.

On the morning of Day 1, urine (first morning void) will be collected for urinary GL-3 and lyso-GB3 determinations followed by administration of an oral dose of 150 mg of AT1001 2 hours prior to the scheduled agalsidase infusion. Subjects will fast for at least 2 hours before and 2 hours after AT1001 administration. In Period 2, each subject will receive the identical agalsidase dose administered in Period 1 as an infusion using an infusion pump. The agalsidase infusion will be initiated 2 hours after administration of the AT1001 dose.

Blood samples for pharmacokinetic and pharmacodynamic analysis will be collected before dosing AT1001 and at 1 hour after administration of AT1001. Additional blood samples will be collected immediately before initiation of the agalsidase infusion and over the 24-hour period after initiation of the agalsidase infusion. Plasma and WBC α-Gal A enzyme levels, plasma lyso-GB3, and plasma antibody titer will be determined from the collected blood samples at the times summarized in Table 3 for agalsidase beta and in Table 6 for agalsidase alfa. A 12-lead ECG will be performed at the end of the agalsidase infusion, immediately after collection of the post-infusion blood sample. In addition, vital signs will be collected and adverse events and concomitant medications will be assessed.

On Day 2, a punch skin biopsy will be collected 24 hours after initiation of the previous day's infusion from which α-Gal A enzyme levels will be determined; if sufficient sample is available, skin GL-3 will also be determined. After collection of the last pharmacokinetic

sample and before discharge, the following assessments will be performed: adverse event assessment, concomitant medications, physical exam, weight, vital signs, and clinical laboratory tests (serum chemistry, hematology, a blood sample for plasma and WBC α-Gal A, and urinalysis).

On Day 7, subjects will return to the clinical site and have the following assessments performed: physical exam, vital signs, concomitant medications, and adverse event assessment. A skin biopsy will be collected from which α-Gal A enzyme levels will be determined; if sufficient sample is available, skin GL-3 will also be determined. A blood sample for WBC α-Gal A, plasma enzyme level measurement, and plasma lyso GB-3 will also be collected.

On Day 14, a urine sample (first morning void) for determination of urinary GL-3 and lyso-GB3 excretion will be collected (plasma and urine). A blood sample for WBC α-Gal A and plasma enzyme level determinations will also be collected. In addition, vital signs will be collected and adverse events and concomitant medications will be assessed.

Period 3

After completing all assessments after Period 2, all subjects will receive their next agalsidase infusion on Day 1 following their usual dosing schedule and will have adverse events and concomitant medications assessed; on Day 6, all subjects will return to the clinical site approximately 10 hours before their Period 3 evaluation. Subjects will have the following assessments performed at check-in: adverse event assessment, concomitant medications, physical exam, weight, vital signs, 12-lead ECG, and clinical laboratory tests (serum chemistry, hematology, and urinalysis).

On Day 7, a 150 mg oral dose of AT1001 will be administered. Subjects will fast for at least 2 hours before and 2 hours after AT1001 administration.

Blood samples will be collected before dosing and over the 24-hour period after administration of AT1001. AT1001 concentrations will be measured in all plasma samples (see Table 4 for agalsidase beta and Table 7 for subjects receiving agalsidase alfa for sample collection times). Subjects will also have vital signs, weight, and a 12-lead ECG performed. Adverse events and concomitant medications will be assessed.

On Day 8, after collection of the last pharmacokinetic sample and before discharge, the following assessments will be performed: adverse event assessment, concomitant medications, physical exam, vital signs, weight, and clinical laboratory tests (serum chemistry, hematology, and urinalysis).

Stage 2

Each subject will receive each of the following treatments in the order described below:

Period 1: Agalsidase alone as an infusion

Period 2: A 450 mg oral dose of AT1001 two hours before initiation of an intravenous infusion of agalsidase

Note: the dose of agalsidase administered in Periods 1 and 2 will be identical. Agalsidase alfa will be administered as a 40-minute intravenous infusion; agalsidase beta will be administered as a 2-hour intravenous infusion.

Period 1

Subjects meeting all eligibility criteria will be admitted to the clinical site approximately 10 hours prior to their next scheduled agalsidase infusion (Day -1). Subjects will have the following assessments performed at check-in: adverse event assessment, concomitant medications, physical exam, weight, vital signs, 12-lead ECG, clinical laboratory tests (serum chemistry, hematology, and urinalysis), urine GL-3 and Lyso GB-3, and skin biopsy (punch biopsy for measurement of α-Gal A enzyme levels; if sufficient sample is available, skin GL-3 will also be determined).

On the morning of Day 1, urine (first morning void) will be collected for urinary GL-3 and lyso-GB3 determinations followed by administration of the subject's current agalsidase dose given as an infusion using an infusion pump.

Blood samples for pharmacokinetic and pharmacodynamic analysis will be collected immediately before initiation of agalsidase infusion and over the 24-hour period after initiation of the agalsidase infusion. Plasma and WBC α-Gal A enzyme levels, plasma lyso-GB3, and plasma antibody titer will be determined from the collected blood samples at the times summarized in Table 2 for agalsidase beta and in Table 5 for agalsidase alfa. A 12-lead ECG will be performed at the end of the agalsidase infusion, immediately after collection of the post-infusion blood sample. In addition, vital signs will be collected and adverse events and concomitant medications will be assessed.

On Day 2, a punch skin biopsy will be collected 24 hours after initiation of the previous day's infusion from which α-Gal A enzyme levels will be determined; if sufficient sample is available, skin GL-3 will also be determined. After collection of the last pharmacokinetic sample and before discharge, the following assessments will be performed: adverse event assessment, concomitant medications, physical exam, weight, vital signs, and clinical laboratory tests (serum chemistry, hematology, and urinalysis).

On Day 7, subjects will return to the clinical site and have the following assessments performed: vital signs, concomitant medications, and adverse event assessment. A skin biopsy will be collected from which α-Gal A enzyme levels will be determined; if sufficient sample is available, skin GL-3 will also be determined. A blood sample for WBC α-Gal A, and plasma enzyme level measurement, and plasma lyso GB-3 will also be collected.

On Day 14, a urine sample (first morning void) for determination of urinary GL-3 and lyso-GB3) excretion will be collected. A blood sample for WBC α-Gal A, and plasma enzyme level determinations, and plasma lyso GB-3 will also be collected. In addition, vital signs will be collected and adverse events and concomitant medications will be assessed.

Period 2

Subjects will be re-admitted to the clinical site approximately 10 hours prior to their next scheduled agalsidase infusion (Day -1). Subjects will have the following assessments performed at check-in: adverse event assessment, concomitant medications, physical exam, weight, vital signs, 12-lead ECG, and clinical laboratory tests (serum chemistry, hematology, and urinalysis).

On the morning of Day 1, urine (first morning void) will be collected for urinary GL-3 and lyso-GB3 determinations followed by administration of an oral dose of 450 mg of AT1001 2 hours prior to the scheduled agalsidase infusion. Subjects will fast for at least 2 hours before

and 2 hours after AT1001 administration. In Period 2, each subject will receive the identical agalsidase dose administered in Period 1 as an infusion using an infusion pump. The agalsidase infusion will be initiated 2 hours after administration of the AT1001 dose.

Blood samples for pharmacokinetic and pharmacodynamic analysis will be collected before dosing of AT1001 and at 1 hour after administration of AT1001. Additional blood samples will be collected immediately before initiation of the agalsidase infusion and over the 24-hour period after initiation of the agalsidase infusion. Plasma and WBC α-Gal A enzyme levels, plasma lyso-GB3, and plasma antibody titer will be determined from the collected blood samples at the times summarized in Table 3 for agalsidase beta and Table 6 for agalsidase alfa. A 12-lead ECG will be performed at the end of the agalsidase infusion, immediately after collection of the post-infusion blood sample. In addition, vital signs will be collected and adverse events and concomitant medications will be assessed

On Day 2, a punch skin biopsy will be collected 24 hours after initiation of the previous day's infusion from which α-Gal A enzyme levels will be determined; if sufficient sample is available, skin GL-3 will also be determined. After collection of the last pharmacokinetic sample and before discharge, the following assessments will be performed: adverse event assessment, concomitant medications, physical exam, weight, vital signs, and clinical laboratory tests (serum chemistry, hematology, and urinalysis). A blood sample for WBC and plasma α-Gal A activity will also be collected.

On Day 7, subjects will return to the clinical site and have the following assessments performed: vital signs, physical exam, concomitant medications, and adverse event assessment. A skin biopsy will be collected from which α-Gal A enzyme levels will be determined. A blood sample for WBC α-Gal A, plasma lyso GB-3, and plasma enzyme level measurement will also be collected.

On Day 14, a urine collection (first morning void) for determination of urinary GL-3 and plasma lyso-GB3 excretion will be performed. A blood sample for WBC α-Gal A, plasma lyso GB-3, and plasma enzyme level determinations will also be collected. In addition, vital signs will be collected and adverse events and concomitant medications will be assessed.

Follow-up Visit

Subjects who complete all periods of each dose level or prematurely discontinue will be asked to return for a safety follow up visit, 1 month after the last treatment or last study visit (whichever is later), during which the following assessments will be performed: physical examination, concomitant medication assessment, vital signs, weight, 12-lead ECG, clinical laboratory tests (hematology, serum chemistry (including eGFR), and urinalysis), WBC α-Gal A activity, urine GL-3 and lyso-GB3 (assessed using first morning urine), and adverse event assessment.

Unscheduled Visit

To ensure subject safety, if deemed necessary the investigator can perform an unscheduled visit. If any laboratory assessments, needed immediately for clinical management purposes, are tested locally it is recommended that duplicate samples also be sent for central laboratory analysis. The date and reason for the visit, in addition to information collected from procedures performed, are to be captured in the subject's source notes and Case Report Form (CRF).

Eligibility Criteria

An initial evaluation of eligibility criteria will occur at Screening; waivers of inclusion/exclusion criteria will not be allowed.

Inclusion criteria:

1. Male, diagnosed with Fabry disease and between 18 and 65 years of age, inclusive
2. Body Mass Index (BMI) between 18-35
3. Subject initiated treatment with agalsidase at least 1 month prior to Screening, and has received at least two infusions, before the Screening Visit
4. Subject's dose level, dosing regimen and form (i.e., alfa or beta) of agalsidase have been stable (stable dose defined as not varying by more than ± 20%) for at least 1 month before Screening Visit
5. Subject has a estimated creatinine clearance ≥ 50 mL/min at Screening; creatinine clearance to be estimated using the 4-parameter Modification of Diet in Renal Disease (MDRD) equation:

   eGFR (mL/min/1.73 m^2) = 186 x (Serum creatinine (S_{cr}))$^{-1.154}$ x (Age)$^{-0.203}$ x (0.742 if female) x (1.212 if African-American)
6. Subject agrees to use medically accepted methods of contraception during the study and for 30 days after study completion
7. Subject is willing and able to provide written informed consent

Exclusion criteria:

1. Subject has had a documented transient ischemic attack, ischemic stroke, unstable angina, or myocardial infarction within the 3 months before Screening
2. Subject has clinically significant (CS) unstable cardiac disease (e.g., cardiac disease requiring active management, such as symptomatic arrhythmia, unstable angina, or NYHA class III or IV congestive heart failure)
3. Subject has a history of allergy or sensitivity to study drug (including excipients) or other iminosugars (e.g., miglustat, miglitol)
4. Subject requires a concomitant medication prohibited by the protocol: Glyset® (miglitol), or Zavesca® (miglustat)
5. Any investigational/experimental drug or device within 30 days of Screening, except for use of investigational Enzyme Replacement Therapy for Fabry disease
6. ~~Subject is currently being treated with or has previously received AT1001~~

   Protocol Amendment 4.0, version date November 2, 2011, eliminates Exclusion Crterion 6. The numerical reference of the other Exclusion Criteria will remain as before in prior versions of the protocol.
7. Subject has any intercurrent illness or condition that may preclude the subject from fulfilling the protocol requirements or suggests to the investigator that the potential subject may have an unacceptable risk by participating in this study

**Pharmacokinetic Parameters**
Non-compartmental pharmacokinetic parameters of the area under the plasma concentration time curve (AUC) AUC_{0-t}, $AUC_{infinity}$, maximum plasma concentration (C_{max}), time to maximum plasma concentration (t_{max}), elimination rate constant (k_{el}) and half-life will be calculated from plasma AT1001 concentrations and α-Gal A enzyme levels. Pharmacokinetic parameters will be summarized by treatment using descriptive statistics. The AUC_{0-t}, $AUC_{infinity}$ ratios for each compound alone to the respective compound in combination will be calculated. Pharmacokinetic and pharmacodynamic data for those subjects receiving agalsidase alfa and agalsidase beta will be analyzed separately.
**Statistical Procedures**<br><br>Descriptive statistics (N, mean, standard deviation, and coefficient of variation, standard error, median, minimum and maximum) will be provided as appropriate. The effect of a compound on the co-administered compound will be evaluated by calculation of the individual (by subject) AUC and C_{max} ratios as follows:<br><br>$$\text{AUC Ratio} = \frac{AUC_{infinity\ (combination)}}{AUC_{infinity(alone)}}$$<br><br>$$\text{Cmax Ratio} = \frac{C_{max\ (combination)}}{C_{max(alone)}}$$<br><br>The AUC and C_{max} ratios will be expressed as a mean of the individual ratios and 90% confidence interval for the mean.<br><br>Pharmacokinetic and pharmacodynamic data for those subjects receiving agalsidase alfa and agalsidase beta will be analyzed separately. Results will be presented in tabular and graphic forms, as appropriate. All subjects who are dosed with study medication and have sufficient data to generate reliable pharmacokinetic parameters will be included in the safety and pharmacokinetic analysis.

Figure 1: Study Schematic

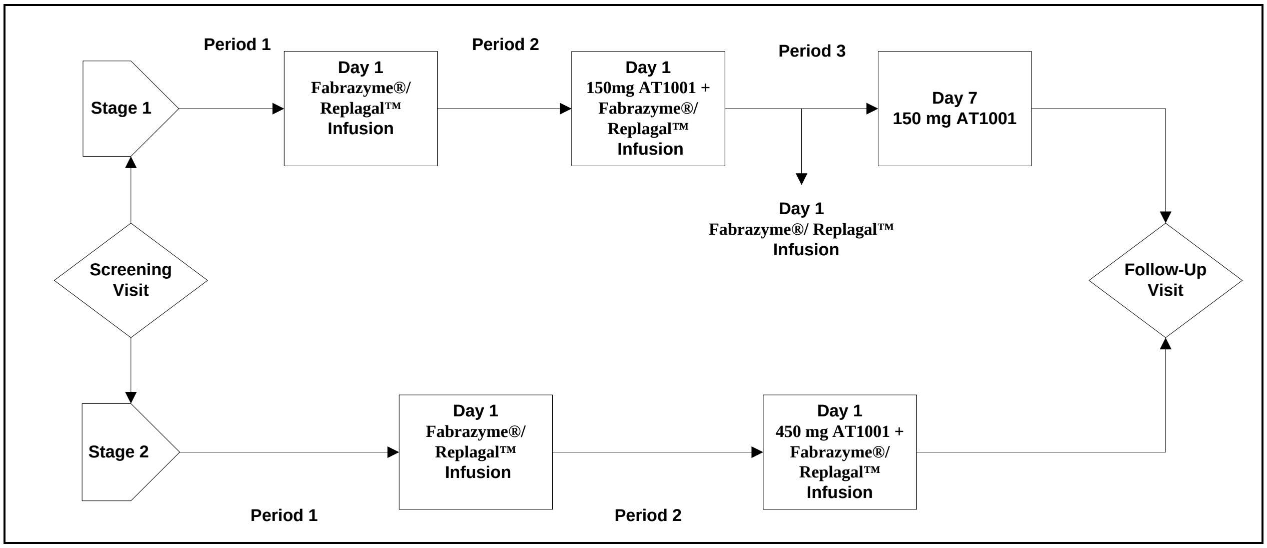

Table 1: Schedule of Assessments (Stages 1 and 2)

	Screening	Period 1 (Stages 1 and 2)					Period 2 (Stages 1 and 2)					Period 3 (Stage 1 only)				Follow Up
**Activity**	**Within 28 days of Enrollment**	**Day -1**	**Day 1**	**Day 2**	**Day 7**	**Day 14**	**Day -1[1]**	**Day 1**	**Day 2**	**Day 7**	**Day 14[1]**	**Day 1**	**Day 6**	**Day 7**	**Day 8**	**1 month after last dose of study medication**
Informed Consent	X															
Medical History and Demographic Data	X															
Physical Exam	X	X		X	X		X		X	X			X		X	**X**
ECG (12-lead)	X	X	X				X	X					X	X		**X**
Vital Signs[2]	X	X	X	X	X	X	X	X	X	X	X		X	X	X	**X**
Hematology	X	X		X			X		X				X		X	**X**
Urinalysis	X	X		X			X		X				X		X	**X**
Serum Chemistry	X	X		X			X		X				X		X	**X**
Height	X															
Weight	X	X		X			X		X				X	X	X	**X**
Coagulation Profile	**X**															
Record of Concomitant Medication	X	←--------------------X--------------------→					←--------------------X--------------------→					←--------------X--------------→				X
eGFR	X															X
Urine GL-3/Lyso-GB3[3]		X	X			X	X	X			X					X
Plasma Lyso-GB3			X		X	X		X		X	X					
Check-in to the clinic		X					X						X			
Drug Dose - Agalsidase			X[4]					X[4]				X[4]				
Drug Dose – AT1001								X						X		
PK Blood Sampling[5]			X	X	X	X		X	X	X	X			X	X	

Table 1: Schedule of Assessments (Stages 1 and 2) (Continued)

Activity	Screening	Period 1 (Stages 1 and 2)					Period 2 (Stages 1 and 2)					Period 3 (Stage 1 only)				Follow Up
	Within 28 days of Enrollment	Day -1	Day 1	Day 2	Day 7	Day 14	Day -1[1]	Day 1	Day 2	Day 7	Day 14[1]	Day 1	Day 6	Day 7	Day 8	1 month after last dose of study medication
Skin Biopsy[6]		X		X	X				X	X						
WBC Collection			X	X	X	X		X	X	X	X					**X**
Antibody Titer[5]			X					X								
Adverse Events		←--------------------X--------------------→					←--------------------X--------------------→					←--------------X--------------→				X

[1]For subjects on a bi-weekly infusion schedule, Day -1 of Period 2 may overlap with Day 14 of Period 1. Day 14 of Period 2 may overlap with Day 1 of Period 3. All procedures listed are to be performed once and transcribed into the applicable CRF.
[2]Vital signs include temperature, blood pressure, heart rate and respiration.
[3]First morning void will be collected for urine GL-3 and lyso GB-3 determination.
[4]Outpatient administration of agalsidase (Fabrazyme® or Replagal™). In Period 2, each subject should receive the identical dose and infusion rate of agalsidase as administered in Period 1.
[5]Sample collection times and analytes are summarized in Table 2, Table 3, Table 4, Table 5, Table 6, and Table 7.

[6]If sufficient sample is available, skin GL-3 will also be measured.

FABRAZYME® (agalsidase beta) Blood Collection Times

Table 2: FABRAZYME® (agalsidase beta) – Period 1, Stage 1 or 2 Blood Collection Times

Study Day	Time (hr)[1]	Blood for plasma α-Gal-A Activity and Western Blot	Blood Draw for			
			Plasma AT1001	WBC α-Gal-A Activity	Plasma IG Titer	Plasma Lyso-GB3
**Period 1, Stage 1 or 2**						
1	0 (pre-infusion)[2]	X		X	X	X
1	0.5	X				
1	1	X				
1	1.5	X				
1	2	X		X		
1	2.5	X				
1	3	X				
1	4	X		X		
1	5	X				
1	6	X				
1	7	X				
1	8	X				
1	12	X				
2	24	X		X		
7	Record Time	X		X		X
14	Record Time	X		X		X

[1] Time is always based on initiation of infusion.
[2] Time 0 is to be collected immediately prior to initiation of the infusion.

Table 3: FABRAZYME® (agalsidase beta) – Period 2, Stage 1 or 2 Blood Collection Times

Study Day	Time (hr)[1]	Blood Draw for plasma α-Gal-A Activity and Western Blot	Blood Draw for			
			Plasma AT1001	WBC α-Gal-A Activity	Plasma IG Titer	Plasma Lyso-GB3
**Period 2, Stage 1, or 2**						
1	-2 (pre-AT1001)	X	X	X	X	X
1	-1		X			
1	0 (pre-infusion)[2]	X	X			
1	0.5	X				
1	1	X	X			
1	1.5	X				
1	2	X	X	X		
1	2.5	X				
1	3	X	X			
1	4	X	X	X		
1	5	X	X			
1	6	X	X			
1	7	X				
1	8	X	X			
1	10		X			
1	12	X				
2	24	X	X	X		
7	Record Time	X		X		X
14	Record Time	X		X		X

[1] Time is always based on initiation of infusion.
[2] Time 0 is to be collected immediately prior to initiation of the infusion.

Table 4: FABRAZYME® (agalsidase beta) – Period 3, Stage 1 ONLY Blood Collection Times

Study Day[1]	Time (hr)	Plasma AT1001
7	0 (pre-AT1001)	X
7	1	X
7	2	X
7	3	X
7	4	X
7	5	X
7	6	X
7	7	X
7	8	X
7	10	X
7	12	X
8	24	X

[1] On Study Day 1 of Period 3, subjects will receive their usual ERT infusion and return on Day 6 for check-in assessments, with AT1001 dosing and PK sampling beginning on Day 7.

REPLAGAL™ (agalsidase alfa) Blood Collection Times

Table 5: REPLAGAL™ (agalsidase alfa) – Period 1, Stage 1 or 2 Blood Collection Times

Study Day	Time (hr)[1]	Blood for plasma α-Gal-A Activity and Western Blot	Blood Draw for			
			Plasma AT1001	WBC α-Gal-A Activity	Plasma IG Titer	Plasma Lyso-GB3
**Period 1, Stage 1 or 2**						
1	0 (pre-infusion)[2]	X		X	X	X
1	0.33 (20 min)	X				
1	0.66 (40 min)	X				
1	1	X				
1	1.5	X				
1	2	X		X		
1	3	X				
1	4	X		X		
1	5	X				
1	6	X				
1	7	X				
1	8	X				
1	12	X				
2	24	X		X		
7	Record Time	X		X		X
14	Record Time	X		X		X

[1] Time is always based on initiation of infusion.
[2] Time 0 is to be collected immediately prior to initiation of the infusion.

Table 6: REPLAGAL™ (agalsidase alfa) – Period 2, Stage 1 or 2 Blood Collection Times

Study Day	Time (hr)[1]	Blood Draw for plasma α-Gal-A Activity and Western Blot	Blood Draw for			
			Plasma AT1001	WBC α-Gal-A Activity	Plasma IG Titer	Plasma Lyso-GB3
**Period 2, Stage 1 or 2**						
1	-2 (pre-AT1001)	X	X	X	X	X
1	-1		X			
1	0 (pre-infusion)[2]	X	X			
1	0.33 (20 min)	X				
1	0.66 (40 min)	X				
1	1	X	X			
1	1.5	X				
1	2	X	X	X		
1	3	X	X			
1	4	X	X	X		
1	5	X	X			
1	6	X	X			
1	7	X				
1	8	X	X			
1	10		X			
1	12	X				
2	24	X	X	X		
7	Record Time	X		X		X
14	Record Time	X		X		X

[1]Time is always based on initiation of infusion.
[2]Time 0 is to be collected immediately prior to initiation of the infusion.

Table 7: REPLAGAL™ (agalsidase alfa) – Period 3, Stage 1 ONLY Blood Collection Times

Study Day[1]	Time (hr)	Plasma AT1001
7	0 (pre-AT1001)	X
7	1	X
7	2	X
7	3	X
7	4	X
7	5	X
7	6	X
7	7	X
7	8	X
7	10	X
7	12	X
8	24	X

[1] On Study Day 1 of Period 3, subjects will receive their usual ERT infusion and return on Day 6 for check-in assessments, with AT1001 dosing and PK sampling beginning on Day 7.

Table 8: Pharmacokinetic Sampling Time Windows

PK Sampling - Nominal Time	Time Window[1]
>0 – 1.0 hour	± 1 minute
>1 – 2.0 hours	± 2 minutes
>2 – 8.0 hours	± 5 minutes
>8 – 24.0 hours	± 15 minutes

[1] Actual time is to be recorded.

LIST OF ABBREVIATIONS

4-MUG	4-methylumbelliferone glucuronide
α-Gal A	α-galactosidase A
aPTT	Activated Partial Thromboplastin Time
AE	adverse event
ALT	alanine aminotransferase
AST	aspartate aminotransferase
ATC	Anatomical Therapeutic Chemical
AUC	area under the plasma concentration time curve
AT1001	migalastat hydrochloride
BID	twice a day
BMI	body mass index
CFR	US Code of Federal Regulations
C_{max}	Maximum plasma concentration
Con A	concanavalin A
CRF	case report form
CS	clinically significant
eGFR	estimated glomerular filtration rate
EOT	end of treatment
ER	endoplasmic reticulum
ERT	enzyme replacement therapy
ECG	electrocardiogram
FDA	Food and Drug Administration
GCP	Good Clinical Practice
GL-3	globotriaosylceramide
GLA	gene encoding α-galactosidase A
ICH	International Conference on Harmonisation
IDMS	Isotope Dilution Mass Spectrometry
IEC	International Ethics Committee
IFG	isofagomine
IgE	Immunoglobulin E
IgG	Immunoglobulin G
INR	International Normalized Ratio
IRB	Institutional Review Board
IV	Intravenous
k_{el}	Elimination rate constant
LC-MS/MS	Liquid Chromatography – Tandem Mass Spectrometry
Lyso-GB3	globotriaosylsphingosine
MDRD	Modification of Diet in Renal Disease

MedDRA	Medical Dictionary for Regulatory Activities
NB-DNJ	N-butyldeoxynojirimycin
NCS	not clinically significant
PD	Pharmacodynamic(s)
PK	pharmacokinetic(s)
PT	Preferred term
rhα-Gal A	Recombinant human α-Gal A
SAE	serious adverse event
S_{cr}	serum creatinine
SOC	system organ class
SUSAR	suspected unexpected serious adverse reaction
T_m	melting temperature
T_{max}	Time of maximum plasma concentration
WBC	white blood cell
WHO	World Health Organization

3. INTRODUCTION AND STUDY RATIONALE

3.1. Fabry Disease

Fabry disease is a progressive, X-linked inborn error of glycospingolipid metabolism caused by a deficiency in the lysosomal enzyme α-Gal A as a result of mutations in the α-Gal A gene,*GLA*. Despite being an X-linked disorder, females may express varying degrees of clinical manifestations. Fabry is a rare disease with incidence estimated between 1 in 40,000 males[1] to 1 in 117,000 in the general population.[2] Moreover, there are variants of later-onset phenotype of Fabry disease that may be under-diagnosed, as they do not present with classical signs and symptoms. This, and the study of newborn screening for Fabry disease[3] suggests that the actual incidence of Fabry disease may be higher than currently estimated.

Clinical manifestation of the disease may correlate with residual α-Gal A levels. Untreated, life expectancy in Fabry patients is reduced and death usually occurs in the fourth or fifth decade because of vascular disease affecting the kidneys, heart and/or central nervous system.

The enzyme deficiency leads to intracellular accumulation of the substrate, globotriaosylceramide (GL-3) in the vascular endothelium and visceral tissues throughout the body.

Gradual deterioration of renal function and the development of azotemia, due to glycospingolipid deposition, usually occur in the third to fifth decades of life, but can occur as early as in the second decade.[4] Renal lesions are found in both hemizygous (male) and heterozygous (female) patients.[5]

Cardiac disease occurs in most males and many females. Early cardiac findings include left ventricular enlargement, valvular involvement and conduction abnormalities. Mitral insufficiency is the most frequent valvular lesion typically present in childhood or adolescence.[6, 7]

Cerebrovascular manifestations result primarily from multifocal small-vessel involvement and may include thromboses, transient ischemic attacks, basilar artery ischemia and aneurysm, seizures, hemiplegia, hemianesthesia, aphasia, labyrinthine disorders, or cerebral hemorrhages. Average age of onset of cerebrovascular manifestations is 33.8 years.[1] Personality change and psychotic behavior may manifest with increasing age.[8]

The current approved treatment for Fabry disease is enzyme replacement therapy (ERT). Two products are currently available for the treatment of Fabry disease: agalsidase alfa (Replagal™, Shire Human Genetic Therapies), marketed outside the United States, and agalsidase beta (Fabrazyme®; Genzyme Corporation), marketed globally. These two forms of ERT are intended to compensate for a patient's inadequate α-Gal A activity with a recombinant form of the enzyme, administered intravenously. While ERT is effective in many settings, the treatment also has limitations. ERT has not been demonstrated to decrease the risk of stroke, cardiac muscle responds slowly, and GL-3 elimination from some of the cell types of the kidneys is limited. Some patients develop immune reactions to ERT.

3.2. AT1001

Fabry disease is a lysosomal storage disorder resulting from a deficiency in the enzyme α Gal A. The deficiency of α-Gal A in Fabry patients is caused by inherited genetic mutations. Certain of these mutations cause changes in the amino acid sequence that may result in the production of α-Gal A with reduced stability that does not fold into its correct three-dimensional shape.[9] Although mutant α-Gal A produced in patient cells often retains the potential for some level of biological activity, the cell's quality control mechanisms recognize and retain misfolded or unstable α-Gal A targeting it in the endoplasmic reticulum (ER) for degradation and elimination. Consequently, little or no α-Gal A moves to the lysosome, where it normally breaks down GL-3. This leads to accumulation of GL-3 in cells, which is believed to contribute to most of the complications associated with Fabry disease. [1, 10, 11]

AT1001 is designed to act as a pharmacological chaperone for α-Gal A by selectively binding to the enzyme, thereby increasing its stability and helping the enzyme fold into its correct three-dimensional shape.[12, 13] This stabilization of α-Gal A allows the cell's quality control mechanisms to recognize the enzyme as properly folded so that trafficking of the enzyme to the lysosome is increased, enabling it to carry out its intended biological function, the metabolism of GL-3. As a result of restoring the proper trafficking of α-Gal A from the ER to the lysosome, AT1001 also reduces the accumulation of misfolded protein in the ER, which may alleviate stress on cells and some inflammatory-like responses that may be contributing factors in Fabry disease.

Multiple in vitro and in vivo preclinical studies of AT1001 have been conducted. AT1001 has been shown to increase the amount of intracellular α-Gal A protein and to enhance transport of mutant enzyme to the lysosome.[14-17]

3.2.1. Nonclinical Summary

To support clinical studies, appropriate pharmacokinetic, toxicokinetic, biodistribution, single and/or multiple dose toxicology, and male and female reproductive studies were performed in rats, dogs, and monkeys. The nonclinical studies were designed to evaluate the plasma clearance and tissue distribution of AT1001, screen for unexpected toxicity, to provide support for the clinical dose regimens, and provide data for use in monitoring the safety of AT1001 in clinical studies.

In summary, the only significant toxicity identified in the completed nonclinical studies of AT1001 has been a reversible impairment of male fertility in rats. The safety profile of AT1001 supports the continued clinical evaluation of this product.

Please refer to the current AT1001 Investigator's Brochure for further details on the nonclinical studies.

3.2.2. Clinical Summary

Four Phase 1 clinical studies were conducted to evaluate the safety, tolerability, and pharmacokinetic properties of AT1001 in healthy male volunteers, one Phase 1 study was conducted to evaluate the effects of AT1001 on QTc intervals, and four Phase 2 studies were conducted to evaluate safety, pharmacokinetics, and pharmacodynamics in adult patients with Fabry disease. A long-term Phase 2 extension study and a Phase 3 study are currently ongoing.

In the four completed Phase 1 studies, 51 healthy male volunteers received AT1001 and 12 received placebo. The AT1001 doses evaluated included single doses of 25, 75, 225, or 675 mg (solution), twice daily doses of 50 or 150 mg (solution) for 7 days, or doses of 100 mg (solution or capsules) in a 3-way crossover study. AT1001 was generally safe and well tolerated in healthy male subjects. There were no deaths, no SAEs, and no discontinuations due to AEs.

Study AT1001-010 was conducted to evaluate the effects of single doses of AT1001 on QTc intervals. AT1001 at single oral doses of 150 mg and 1250 mg was not associated with repolarization changes.

The AT1001 Phase 2 development program comprised four clinical trials (FAB-CL-201, FAB-CL-202, FAB-CL-203, and FAB-CL-204) in male and female patients with Fabry disease that evaluated several different doses and dosing regimens. Clinical trial FAB-CL-205 is an ongoing open-label, long-term extension study for all subjects who have completed any of the four AT1001 Phase 2 clinical trials.

The results of the Phase 1 and 2 studies demonstrate that AT1001 was generally well tolerated at all doses evaluated (single doses up to 2000 mg, and repeated doses up to 250 mg twice daily for 2 weeks or 250 mg every other day for up to 1 year or longer).

The ongoing Phase 3 program consists of study AT1001-011, a double blind, randomized, placebo controlled study evaluating the efficacy, safety and pharmacodynamics of AT1001 in patients with Fabry disease having AT1001-responsive *GLA* mutations.

Please refer to the current AT1001 Investigator's Brochure for further details on the clinical studies.

3.3. Agalsidase

Agalsidase alfa and agalsidase beta are two forms of ERT intended to address the enzyme deficiency in Fabry disease by providing the body with an exogenous enzyme source. ERT has been demonstrated to reduce GL-3 deposition in capillary endothelium of the kidney and certain other cell types.[18-21]

3.3.1. Agalsidase alfa

The recommended dosage of agalsidase alfa is 0.2 mg/kg body weight infused every 2 weeks as an intravenous infusion.[22] A 10-week study was conducted in ERT naïve adult males Fabry patients to evaluate the pharmacokinetics and pharmacodynamics of agalsidase alfa. The mean half life after administration of doses ranging from 0.1 to 0.4 mg/kg of agalsidase alfa was 56-76 minutes with no significant association between dose and half life, clearance or volume of distribution. The AUC was linearly proportional to dose over this dose range. Plasma GL-3 levels were reduced in all dose groups by approximately 50%; the reduction was independent of dose and dosing frequency. Two of 18 patients became IgG positive during the study. No Immunoglobulin E (IgE) antibodies were detected in any patient during the study.[23]

Please refer to the current Replagal™ (agalsidase alfa) Prescribing Information for further details.

3.3.2. Agalsidase beta

The recommended dosage of agalsidase beta is 1 mg/kg body weight infused every 2 weeks as an intravenous infusion. The manufacturer of agalsidase beta has announced a drug shortage, the only ERT approved in the US for Fabry disease. As a result, agalsidase beta is currently rationed and patients typically receive a reduced dose of the enzyme and/or an extended dosing interval (*i.e.,* greater than 2 weeks between doses).

Agalsidase beta exhibits non-linear pharmacokinetics with exposure (AUC) values increasing and clearance decreasing disproportionally with increase in dose. AUC values increased approximately 6-fold and 8-fold when doses were increased from 0.3 mg/kg to 1 mg/kg and from 1 mg/kg to 3 mg/kg, respectively.[18] The elimination half-life of agalsidase beta in adult patients after doses ranging from 0.3 mg/kg to 3 mg/kg was dose dependent and ranged from 45 to 100 minutes.[24]

IgG antibodies to agalsidase beta developed in 79% of adult patients and 69% of pediatric patients treated with agalsidase in clinical studies; the majority of patients who developed IgG antibodies did so within the first 3 months of exposure. Males, particularly those with low residual α-Gal A levels, were more likely to develop IgG antibodies than males with higher residual levels or in females. IgG seroconversion in pediatric patients was associated with prolonged half-life of agalsidase.[24] However, in adult patients, identical agalsidase pharmacokinetic profiles were observed before and after seroconversion in one trial;[18] in another trial, maximal agalsidase concentrations and AUC values were reduced up to 26% of baseline values in patients with the highest titers of IgG.[25] The presence of IgG antibodies to agalsidase has been reported to decrease activity of the enzyme.[26]

Please refer to the current Fabrazyme® (agalsidase beta) Prescribing Information for further details.

3.4. Effect of AT1001 on the Pharmacokinetics and Pharmacodynamics of α-Gal A

Pharmacological chaperones have been demonstrated to have beneficial effects on several exogenous recombinant enzymes used to treat lysosomal storage disorders, including improving physical stability and cellular and tissue uptake of the enzyme. Specifically, the pharmacological chaperones isofagomine (IFG) and N-butyldeoxynojirimycin (NB-DNJ) were shown to increase the in vitro cellular uptake and in vivo tissue uptake of the recombinant enzymes used to treat Gaucher (imiglucerase, Cerezyme; Genzyme Corporation) and Pompe (alglucosidase alfa, Myozyme®; Genzyme Corporation) disease. [27, 28]

Please refer to the current AT1001 Investigator Brochure's for further information.

3.4.1. Effect of AT1001 on Fabrazyme® (agalsidase beta)

AT1001 stabilizes agalsidase beta in vitro and as well as in vivo. It has been demonstrated in vitro that the binding of AT1001 to rhα-Gal A resulted in significant time- and concentration-dependent increases in stabilization of rhα-Gal A at neutral pH as measured by thermal denaturation and by activity. In a neutral pH buffer, rhα-Gal A showed a loss in activity, with a half-life of approximately 3 hours; co-incubation with AT1001 increased the half-life for loss of rhα-Gal A activity to approximately 40 hours.

In the rat, oral administration of 3 mg/kg of AT1001 followed 30 min later by an injection of 10 mg/kg agalsidase beta resulted in a 2.6-fold increase in the plasma half-life of rhα-Gal A and a 2.5-fold and 1.5-fold increase in plasma α-Gal A levels at 60 and 240 minutes, respectively.

In the *GLA* deficient mouse, oral administration of 30, 100 or 300 mg/kg doses of AT1001 30 min prior to and 2 hours after an injection of rhα-Gal A resulted in a dose-dependent increase in tissue α-Gal A levels and a dose-dependent reduction in GL-3 levels in skin, heart, kidney, and plasma compared to administration of rhα-Gal A alone.

Please refer to the current AT1001 Investigator Brochure's for further information.

3.4.2. Effect of AT1001 on Replagal™ (agalsidase alfa)

AT1001 has been shown to stabilize agalsidase alfa both in vitro and in vivo. The effect of AT1001 on the physical stability of agalsidase alfa was evaluated with an in vitro thermal denaturation assay. Using this assay, agalsidase alfa showed a melting temperature (T_m) of approximately 51°C at pH 7.4. However, when 10 μM AT1001 was included in the denaturation reaction, the T_m of agalsidase alfa was substantially increased to 59°C. As expected for a lysosomal enzyme, agalsidase alfa was more stable at low pH (T_m of 58°C at pH 5.2) and exhibited further resistance to heat-denaturation in the presence of 10 μM AT1001 (T_m of 68°C). These data indicate that binding of AT1001 confers a high level of physical stability to agalsidase alfa.

The effect of AT1001 on the rate of clearance of agalsidase alfa from the blood of male Sprague-Dawley rats was also investigated. Animals received vehicle (water) or a single oral gavage of 1, 3, 10, or 30 mg/kg AT1001, followed 30 minutes later by intravenous administration of 0.2 mg/kg agalsidase alfa via bolus tail vein injection. Blood was collected as a function of time and α-Gal A activity was measured in plasma. In the absence of AT1001, α-Gal A activity declined rapidly; pre-administration of AT1001 resulted in a dose-dependent increase in the half life of agalsidase alfa (as measured by α-Gal A activity) of approximately 2-fold and 3-fold, after administration of 3 mg/kg and 30 mg/kg AT1001, respectively, with an approximately 2.5-fold and 1.5-fold increase in plasma α-Gal A levels at 60 and 240 minutes, respectively.

The effect of AT1001 on agalsidase alfa both in vitro and in vivo is comparable to that observed with AT1001 on agalsidase beta.

3.5. Preliminary Safety Evaluation of Co-Administered AT1001 and Fabrazyme® in *GLA* Deficient Mice

A preliminary study in *GLA* deficient mice has been conducted evaluating the safety of co-administered AT1001 and Fabrazyme®. AT1001 was administered three times a week for four weeks at doses of 3 and 30 mg/kg in combination with Fabrazyme® administered intravenously once a week at a dose of 1 mg/kg. There appeared to be no direct drug-related changes in survival, clinical condition or hematology and clinical chemistry parameters observed in male *GLA*-deficient mice co-administered with AT1001 and Fabrazyme®.

For additional details, see the AT1001 Investigator's Brochure.

3.6. Study Rationale

3.6.1. Clinical Rationale

This protocol describes a Phase 2a, multi-site, drug-drug interaction study of AT1001 and agalsidase. This study will be conducted in 2 successive stages that will:

- Stage 1: evaluate the effect of 150 mg AT1001 on agalsidase safety and pharmacokinetics and agalsidase on 150 mg AT1001 safety and pharmacokinetics
- Stage 2: evaluate the effect of 450 mg AT1001 on agalsidase safety and pharmacokinetics.

This study will provide drug-drug interaction information after co-administration of AT1001 and agalsidase. In addition, this study is intended as a pilot study in patients with Fabry disease, to both validate the migalastat-agalsidase co-administration animal studies and to provide initial proof of concept data in patients with Fabry disease, on whether AT1001 has the potential to improve the pharmacokinetic properties of agalsidase. Patients receiving either agalsidase alfa (Replagal™) or agalsidase beta (Fabrazyme®) will be eligible to participate in this study. Male subjects between 18 and 65 years of age who have been receiving a stable dose (0.3-1.0 mg/kg) of agalsidase beta or (≥ 0.2 mg/kg) of agalsidase alfa or at least one month before study entry and who meet all other eligibility criteria will be enrolled into the study. The results of this study may support further development of AT1001 in combination with Enzyme Replacement Therapy.

3.6.2. AT1001 Dose Rationale

The administration of AT1001 with agalsidase to *GLA* knock-out mice was associated with a greater increase in tissue α-Gal A enzyme levels and greater reduction of GL-3 compared to administration of agalsidase alone (see AT1001 Investigator's Brochure for details). Similar studies involving the administration of migalastat prior to agalsidase infusion in Sprague-Dawley rats resulted in a 2.6-fold increase in α-Gal A half-life in plasma compared to agalsidase alone. The peak plasma concentrations of migalastat attained were similar to or lower than those that would be achieved by 150 mg and 450 mg doses in humans. In a preliminary evaluation of the safety of co-administered AT1001 (3 times a week) and Fabrazyme® (once a week) for 28 days in *GLA* deficient mice there appeared to be no direct drug-related changes in survival, clinical condition or hematology and clinical chemistry parameters observed in male *GLA*-deficient mice associated with co-administration of AT1001 and Fabrazyme®.

The safety, tolerability and pharmacokinetics of AT1001 has been studied in healthy volunteers after single doses up to 2000 mg and after multiple daily doses up to 300 mg administered as 150 mg twice a day (BID) and in Fabry patients at daily doses up to 500 mg, administered as 250 mg BID. AT1001 was well tolerated in healthy volunteers at doses up to 2000 mg and in Fabry patients at doses up to 250 mg twice daily. The most commonly reported treatment-emergent adverse events observed in Fabry patients included headache, arthralgia, diarrhea, and nausea. Most of these were of mild or moderate intensity and not related to study medication. Only one serious adverse event SAE had an association with study drug (the episode of heart block that was considered unlikely related to treatment). Only one AE-related discontinuation, due to hypertension and epistaxis after receiving AT1001 25 mg twice daily for 6 days, has

occurred to date. The investigator deemed the hypertension to be possibly related to study drug and the epistaxis to be unlikely related to study drug. The events resolved without intervention.

Overall, there were no trends or clinically meaningful changes in standard safety assessments that included ECG measurements, vital signs and physical examinations and clinical laboratory assessments. No deaths have been reported.

AT1001 at a dose of 150 mg every other day is currently being evaluated in patients with responsive mutations as a single agent for the treatment of Fabry disease. Repeat dosing with the regimen of 150 mg AT1001 once every other day in humans is associated with a plasma Cmax value of 1500-2000 ng/mL (9-12 μM), a terminal elimination half-life of ranging from 2.5 to 4.5 hours, and no observed accumulation in plasma. This dose has been administered to 36 subjects in the Phase 2 program, appears to be generally well tolerated, and is associated with increases in WBC α-Gal A levels, decreases in the number of GL-3 inclusions in kidney interstitial capillaries, and decreases in urine GL-3 levels in subjects with AT1001-responsive *GLA* mutations.

There will be two AT1001 dose levels evaluated in this study, 150 mg and 450 mg.

The information gained from evaluating the potential for a drug-drug interaction between 150 mg AT1001 monotherapy and ERT will be used to inform the management of patients with Fabry disease who may be exposed to both agents at the same time in the course of their treatment.

The administration of 150 and 450 mg AT1001 in conjunction with agalsidase is intended to provide information on the dose-response of any observed effect of AT1001 on agalsidase pharmacokinetics.

The presence of a positive interaction between 150 mg and/or 450 mg of AT1001 on agalsidase will provide the basis to support further clinical evaluation of the co-administration of AT1001 with Enzyme Replacement Therapy.

4. STUDY OBJECTIVES

4.1.1. Primary Objectives

- To characterize the effects of 150 mg and 450 mg of AT1001 administered 2 hours before administration of agalsidase on the safety and plasma pharmacokinetics of agalsidase in subjects with Fabry Disease
- To characterize the effect of agalsidase on the safety and plasma pharmacokinetics of 150 mg of AT1001 administered 2 hours before administration of agalsidase in subjects with Fabry Disease

4.1.2. Secondary Objective

- To characterize the effect of 150 mg and 450 mg AT1001 on the distribution of α-Gal A to skin after administration of agalsidase

4.2. Study Endpoints

4.2.1. Primary Endpoints

- AT1001 plasma pharmacokinetic parameter values after administration of a single oral dose of AT1001 alone and in combination with agalsidase
- Agalsidase plasma pharmacokinetic parameter values by measurement of α-Gal A enzyme levels and protein levels after agalsidase infusion alone and in combination with oral AT1001
- Safety variables: adverse events, clinical laboratory tests, 12-Lead ECGs, physical examinations, vital signs, and infusion reactions

4.2.2. Secondary Endpoint

- Distribution of agalsidase to skin after agalsidase alone and in combination with AT1001 24 hours, and 7 days after dosing by measuring α-Gal A levels and protein levels

4.2.3. Exploratory Endpoints

- Urinary GL-3 excretion before and 14 days after each agalsidase dose
- WBC α-Gal A enzyme levels, determined before initiation of the agalsidase infusion and at 2, 4, and 24 hours and 7 and 14 days after dosing
- Antibody titer (IgG) before initiation of an infusion of agalsidase
- Plasma lyso-GB3 concentrations and urinary excretion of lyso-GB3

5. STUDY DESIGN

This open-label study will consist of two stages (see Figure 1). Stage 1 will consist of screening and a three-period study to evaluate the effect of 150 mg AT1001 on the pharmacokinetics and safety of agalsidase and the effect of agalsidase on the pharmacokinetics of 150 mg AT1001. Stage 2 will consist of screening and a two-period study to evaluate the effect of 450 mg AT1001 on the pharmacokinetics of agalsidase (the effect of agalsidase on the pharmacokinetics of a 450 mg dose of AT1001 will not be evaluated).

Male subjects between 18 and 65 years of age who have been receiving a stable dose (0.3-1.0 mg/kg) of agalsidase beta or (≥ 0.2 mg/kg) of agalsidase alfa for at least one month before study entry and who meet all other eligibility criteria will be enrolled into the study. A stable dose of enzyme is defined as a dose not varying by more than ±20%. The first ten to twelve subjects (a minimum of 4 subjects receiving agalsidase alfa; the remaining subjects receiving agalsidase beta) meeting all eligibility criteria will be enrolled into Stage 1. The decision to initiate dosing in Stage 2 will be made by the Amicus Medical Monitor and Investigator(s) after consideration of safety and tolerability information from at least the first 4 subjects having completed Stage 1. Subjects on agalsidase beta will be assigned to Stage 2 after a minimum of 6 subjects complete Stage 1. Subjects on agalsidase alfa will be assigned to Stage 2 after a minimum of four subjects complete Stage 1. At least four subjects will be treated with 450 mg AT1001 co-administered with each form of ERT in Stage 2.

The end of the study will be defined as last subject/last visit. On completion of the study, each subject will be returned to the most appropriate treatment and care recommended by their routine physician.

5.1. Randomization and Blinding

This will be an open-label study. Subjects completing the study screening assessments and meeting all the eligibility requirements will be enrolled into the study and receive their regular scheduled dose of agalsidase in Period 1, dose of agalsidase plus AT1001 in Period 2, and dose of AT1001 alone in Period 3.

6. SELECTION OF SUBJECTS

6.1. Target Population

This study will be conducted in males who have Fabry disease who are currently receiving a stable dose of agalsidase alfa or agalsidase beta every 2 to 4 weeks.

6.2. Number of Subjects

Approximately four to six subjects using each form of ERT at each dose level (i.e., Fabrazyme® + AT1001 150 mg; Fabrazyme® + AT1001 450 mg; Replagal™ + AT1001 150 mg; and Replagal™ + AT1001 450 mg) for a possible total of 24 evaluable subjects.

6.3. Recruitment Strategy

Investigators may enroll subjects from their existing or incoming patients, ask other physicians for referrals of suitable patients, or advertise the study in public media after review and approval by Amicus and Independent Ethics Committee/Institutional Review Board (IEC/IRB)

6.4. Inclusion Criteria

Subjects must meet all of the following criteria at Screening in order to be considered for enrollment into the study (waivers to inclusion/exclusion criteria will not be allowed):

1. Male, diagnosed with Fabry disease and between 18 and 65 years of age, inclusive
2. Body Mass Index (BMI) between 18-35
3. Subject initiated treatment with agalsidase at least 1 month prior to Screening, and has received at least two infusions, before the Screening Visit
4. Subject's dose level, dosing regimen and form (i.e., alfa or beta) of agalsidase have been stable (stable dose defined as not varying by more than ± 20%) for at least 1 month before Screening Visit
5. Subject has a estimated creatinine clearance ≥ 50 mL/min at Screening; creatinine clearance to be estimated using the 4-parameter MDRD equation:

   $$\text{eGFR (mL/min/1.73 m}^2\text{)} = 186 \times (\text{Scr})^{-1.154} \times (\text{Age})^{-0.203} \times (0.742 \text{ if female}) \times (1.212 \text{ if African-American})$$

6. Subject agrees to use medically accepted methods of contraception during the study and for 30 days after study completion
7. Subject is willing and able to provide written informed consent

6.5. Exclusion Criteria

Subject candidates must not be enrolled in the study if they meet any of the following criteria:

1. Subject has had a documented transient ischemic attack, ischemic stroke, unstable angina, or myocardial infarction within the 3 months before Screening

2. Subject has clinically significant unstable cardiac disease (e.g., cardiac disease requiring active management, such as symptomatic arrhythmia, unstable angina, or NYHA class III or IV congestive heart failure)
3. Subject has a history of allergy or sensitivity to study drug (including excipients) or other iminosugars (e.g., miglustat, miglitol)
4. Subject requires a concomitant medication prohibited by the protocol: Glyset® (miglitol), or Zavesca® (miglustat)
5. Any investigational/experimental drug or device within 30 days of Screening, except for use of investigational Enzyme Replacement Therapy for Fabry Disease
6. ~~Subject is currently being treated with or has previously received AT1001~~

   Protocol Amendment 4.0, version date November 2, 2011, eliminates Exclusion Criterion 6. The numerical reference of the other Exclusion Criteria will remain as before in prior versions of the protocol.
7. Subject has any intercurrent illness or condition that may preclude the subject from fulfilling the protocol requirements or suggests to the investigator that the potential subject may have an unacceptable risk by participating in this study

7. STUDY TREATMENT

7.1. Details of Study Treatments

AT1001 (migalastat hydrochloride, 150 mg per capsule) is formulated with magnesium stearate and pregelatinized starch in a white, hard gelatin capsule. AT1001 capsules will be supplied by Amicus. Refer to the Investigator's Brochure for AT1001 for additional information.

Fabrazyme® (agalsidase beta) or Replagal™ (agalsidase alfa) at the patients currently prescribed dose level will be administered as an intravenous infusion using a calibrated infusion pump. Agalsidase will be supplied by the clinical site. Refer to the current Fabrazyme® or Replagal™ Prescribing Information, as appropriate, for additional information.

7.2. Dosage Schedule

Each subject will receive each of the following treatments in the order described below:

Table 9: Treatment Schedule

Stage 1	
**Period 1**	Agalsidase alone as an intravenous infusion
**Period 2**	A single oral dose of 150 mg AT1001 two hours before initiation of an intravenous infusion of agalsidase. Subjects will be required to fast 2 hours before and 2 hours after taking AT1001.
**Period 3**	A single oral dose of 150 mg AT1001 alone. Subjects will be required to fast 2 hours before and 2 hours after taking AT1001.
**Stage 2**	
**Period 1**	Agalsidase alone as an intravenous infusion
**Period 2**	A single oral dose of 450 mg AT1001 two hours before initiation of an intravenous infusion of agalsidase. Subjects will be required to fast 2 hours before and 2 hours after taking AT1001.

Agalsidase alfa (Replagal®) will be administered as a 40-minute infusion; agalsidase beta (Fabrazyme®) will be administered as a 2-hour infusion. Every effort should be made to ensure the duration of infusion during Periods 1 and 2 are the same.

7.3. Dose Escalation

A Steering Committee will be chartered to monitor and evaluate the safety of all subjects in this trial by periodically reviewing summaries of safety data, evaluating risk/benefit where possible, and identifying any clinically relevant trends through completion of each cohort assessing whether it is safe to continue and enroll the next sequential dose level/cohort. The committee will be composed of the Amicus Medical Monitor and the Investigators. The operational and logistical procedures for the committee will be detailed in a charter. Safety data reviewed will include adverse events (including infusion-associated reactions), clinical laboratory tests (hematology, urinalysis, and serum chemistry (including eGFR), 12-lead ECGs, physical examinations, and vital signs.

7.4. Packaging and Labeling

Study medication (AT1001) will be packaged as bulk bottles for open-label administration and will be supplied by Amicus to the study sites as 150 mg capsules. Each bottle will be labeled in conformance to regulatory requirements and where applicable, local laws. All labels will be printed with the following information at a minimum: study identifier, identity of drug, capsule strength and quantity, Sponsor name and contact details (and/or details of a local designee contact), dosing instructions, storage information and other applicable local law statements.

7.5. Supplies and Accountability

Sites will be instructed to store the study medication (AT1001) at room temperature (approximately 15-25°C; 59-77°F) in a secure area, free from environmental extremes, and with restricted access.

The investigator agrees neither to dispense the study medication from, nor store it, at any site(s) other than those listed on the Form Food and Drug Administration (FDA) 1572. The investigator agrees that study medication will be dispensed only to subjects who have provided written informed consent, have met all entry criteria and were randomized.

The investigator, or appropriately assigned designee, will inventory and acknowledge receipt of all shipments of the study medication. The investigator agrees to keep accurate records of the quantities of study medication dispensed and administered to each subject. The study monitor will periodically check the supplies of study medication held at the site to verify accountability of all study medication used and to verify that a final report of drug accountability to the unit dose level is prepared and maintained in the investigator study file. When instructed by the monitor, the investigator agrees to return all original containers of study medication, whether empty, or containing used or unused study medication to the sponsor or their designee.

7.6. Compliance

Administration of study medication (i.e., agalsidase and/or AT1001) at each visit (if the visit occurs on a dosing day) will be supervised by the Investigator or sub-Investigator. Any delegation of this responsibility must follow Section 13.2.

The Investigator may choose to interrupt or discontinue the study medication in case of an AE or for administrative reasons. Any interruption in dosing should be documented in the appropriate CRF.

The Investigator should discontinue the study medication if continued administration of the study medication is believed to be contrary to the best interest of the subject. The reasons for discontinuation must be documented in the CRF.

If the reason for the interruption or discontinuation of the study medication is an AE, an abnormal assessment (e.g., ECG finding), or a laboratory test abnormality, this information will be recorded as an adverse event in the CRF.

If the subject is not compliant with the study medication administration, the Investigator will need to evaluate whether the noncompliance should warrant subject withdrawal from the study.

Subjects who discontinue from the study will be replaced.

8. PRIOR AND CONCOMITANT ILLNESSES AND TREATMENTS

8.1. Prior and Concomitant Illnesses

Illnesses present at the time informed consent is provided are regarded as concomitant illnesses and must be documented in the CRF along with the subject's other medical history.

Illnesses first occurring or detected during the study, and worsening of a concomitant illness during the study, are to be regarded as adverse events and must be documented as such in the CRF (see Section 9).

8.2. Prior and Concomitant Treatments

Concomitant medications taken within 4 weeks prior to screening or at any time throughout the study must be recorded in the CRF, along with the reason for use, dates of administration, dosages and frequency.

Use of the following medications is prohibited at any time throughout the study:

- Glyset® (miglitol)
- Zavesca® (miglustat)
- Investigational/experimental therapy

Should any of the prohibited medications be initiated, all end-of-treatment assessments (i.e., physical examination, 12-Lead ECG and clinical laboratory evaluations) shall be performed and the study medication must be permanently discontinued.

8.3. Description of Study Visits

Informed consent will be obtained before any other study-specific procedures are performed. The screening visit window should be calculated from the date of the first procedure performed after the subject provides written informed consent. If more than 30 days passes between consent and a procedure in the clinic, the subject should be asked provide written agreement to continue participation in the study. The screening visit must occur within 28 days of Period 1 (Day -1) to determine eligibility.

Every effort should be made to perform study visits within the pre-specified visit schedule. In the event of extenuating circumstances, it is best to have a visit performed early or later than the projected time point instead of having a missed visit. A study month is defined as 30 days.

8.3.1. Screening

The following assessments will be performed during screening:

- Review entry criteria
- Medical history (including *GLA* genotype, if known)
- Height and weight
- Concomitant/prior medications
- Physical exam
- Vital signs

- 12-lead ECG
- Clinical laboratory tests (serum chemistry, hematology, coagulation profile, urinalysis)
- eGFR

8.3.2. Stage 1

8.3.2.1. Period 1

Subjects meeting all eligibility criteria will be admitted to the clinical site approximately 10 hours prior to their next scheduled agalsidase infusion. Subjects will have the following assessments performed at check-in (Day -1):

- Adverse event assessment
- Concomitant medications
- Physical examination
- Vital signs
- Weight
- 12-lead ECG
- Clinical laboratory tests (serum chemistry, hematology, and urinalysis)
- Urine GL-3/Lyso GB-3
- Skin biopsy (punch biopsy for measurement of α-Gal A enzyme levels; if sufficient sample is available, skin GL-3 will also be determined)

On the morning of Day 1, urine (first morning void) will be collected for urinary GL-3 and lyso-GB3 determinations followed by administration of the subject’s current agalsidase dose given as an infusion using an infusion pump.

Blood samples for pharmacokinetic and pharmacodynamic analysis will be collected immediately before initiation of the agalsidase infusion and over a 24-hour period after initiation of the agalsidase infusion. Plasma and WBC α-Gal A enzyme levels, plasma lyso-GB3 and plasma antibody titer will be determined from the collected blood samples at the times summarized in Table 2 for agalsidase beta and in Table 5 for agalsidase alfa. A 12-lead ECG will be performed at the end of the agalsidase infusion, immediately after collection of the post-infusion blood sample. Vital signs will be recorded as defined in Section 8.4.9. Adverse events and concomitant medications will also be assessed.

On Day 2, a punch skin biopsy will be collected 24 hours after initiation of the infusion from which α-Gal A enzyme levels will be determined; if sufficient sample is available, skin GL-3 will also be determined. After collection of the last pharmacokinetic sample and before discharge, the following assessments will be performed:

- Adverse event assessment
- Concomitant medications
- Physical examination
- Weight
- Vital signs
- Clinical laboratory tests (serum chemistry, hematology, and urinalysis)
- A blood sample for WBC and plasma α-Gal A levels

On Day 7, subjects will return to the clinical site and have the following assessments performed:

- Adverse event assessment
- Concomitant medications
- Physical examination
- Vital signs
- Skin biopsy (punch biopsy for measurement of α-Gal A enzyme levels; if sufficient sample is available, skin GL-3 will also be determined)
- A blood sample for WBC and plasma α-Gal A levels, and plasma Lyso GB-3

On Day 14, a urine sample (first morning void) for determination of urinary GL-3 and lyso-GB3 excretion will be collected.

A blood sample for WBC and plasma α-Gal A levels, and plasma Lyso-GB3 levels will also be collected. In addition, the following assessments will be performed:

- Adverse event assessment
- Concomitant medications
- Vital signs

For subjects on a bi-weekly infusion schedule, Day -1 of Period 2 may overlap with Day 14 of Period 1.

8.3.2.2. Period 2

Subjects will be re-admitted to the clinical site approximately 10 hours prior to their next scheduled agalsidase infusion. Subjects will have the following assessments performed at check-in (Day -1):

- Adverse event assessment
- Concomitant medications
- Physical exam
- Vital Signs
- Weight
- 12-lead ECG
- Clinical laboratory tests (serum chemistry, hematology, and urinalysis)
- Urine GL-3/Lyso GB-3

On the morning of Day 1, urine (first morning void) will be collected for urinary GL-3 and lyso-GB3 determinations followed by administration of an oral dose of 150 mg of AT1001 2 hours prior to the scheduled agalsidase infusion. Subjects will fast for at least 2 hours before and 2 hours after AT1001 administration. In Period 2, each subject will receive the identical agalsidase dose administered in Period 1 as an infusion using an infusion pump. The agalsidase infusion will be initiated 2 hours after administration of the AT1001 dose.

Blood samples for pharmacokinetic and pharmacodynamic analysis will be collected before dosing AT1001 and at 1 hour after administration of AT1001. Additional blood samples will be collected immediately before initiation of the agalsidase infusion over the 24-hour period after initiation of the agalsidase infusion. Plasma and WBC α-Gal A enzyme levels, plasma lyso-GB3, and plasma antibody titer will be determined from the collected blood samples at the times summarized in Table 3 for agalsidase beta and in Table 6 for agalsidase alfa. A 12-lead ECG will be performed at the end of the agalsidase infusion, immediately after collection of the post-infusion blood sample. Vital signs will be recorded as defined in Section 8.4.9.

In addition, the following assessments will be performed:

- Adverse event assessment
- Concomitant medications

On Day 2, a punch skin biopsy will be collected 24 hours after initiation of the infusion from which α-Gal A enzyme levels will be determined; if sufficient sample is available, skin GL-3 will also be determined. After collection of the last pharmacokinetic sample and before discharge, the following assessments will be performed:

- Adverse event assessment
- Concomitant medications
- Physical examination
- Vital signs
- Weight
- Clinical laboratory tests (serum chemistry, hematology, and urinalysis)
- A blood sample for WBC and plasma α-Gal A levels

On Day 7, subjects will return to the clinical site and have the following assessments performed:

- Adverse event assessment
- Concomitant medications
- Physical examination
- Vital signs
- Skin biopsy (punch biopsy for measurement of α-Gal A enzyme levels; if sufficient sample is available, skin GL-3 will also be determined)
- A blood sample for WBC and plasma α-Gal A levels, and plasma lyso GB-3 levels

On Day 14, a urine sample (first morning void) for determination of urinary GL-3 and lyso-GB3 excretion will be collected.

A blood sample for WBC and plasma α-Gal A levels, and plasma Lyso-GB3 levels will also be collected. In addition, the following assessments will be performed:

- Adverse event assessment
- Concomitant medications
- Vital signs

For subjects on a bi-weekly infusion schedule, Day -1 of Period 2 may overlap with Day 14 of Period 1. Day 14 of Period 2 may overlap with Day 1 of Period 3.

8.3.2.3. Period 3

After completing all assessments after Period 2, all subjects will receive their next agalsidase infusion on Day 1 following their usual dosing schedule and will have adverse events and concomitant medications assessed; on Day 6, all subjects will return to the clinical site approximately 10 hours before their Period 3 evaluation. Subjects will have the following assessments performed at check-in:

- Adverse event assessment
- Concomitant medications
- Physical exam
- Vital signs

- Weight
- 12-lead ECG
- Clinical laboratory tests (serum chemistry, hematology and urinalysis).

On Day 7, a 150 mg oral dose of AT1001 will be administered. Subjects will fast for at least 2 hours before and 2 hours after AT1001 administration. Vital signs and weight will be recorded, and a 12-lead ECG will be performed prior to treatment.

In addition, the following assessments will be performed:

- Adverse event assessment
- Concomitant medications

Blood samples will be collected pre-dose and over a 24-hour period after administration of AT1001. AT1001 concentrations will be measured in all plasma samples for agalsidase beta (Table 4) and for agalsidase alfa (Table 7).

On Day 8, after collection of the last pharmacokinetic sample and before discharge, the following assessments will be performed:

- Adverse event assessment
- Concomitant medications
- Physical exam
- Vital signs
- Weight
- Clinical laboratory tests (serum chemistry, hematology, and urinalysis).

8.3.3. Stage 2

8.3.3.1. Period 1

Subjects meeting all eligibility criteria will be admitted to the clinical site approximately 10 hours prior to their next scheduled agalsidase infusion. Subjects will have the following assessments performed at check-in (Day -1):

- Adverse event assessment
- Concomitant medications
- Physical examination
- Vital signs
- Weight
- 12-lead ECG
- Clinical laboratory tests (serum chemistry, hematology, and urinalysis)
- Skin biopsy (punch biopsy for measurement of α-Gal A enzyme levels; if sufficient sample is available, skin GL-3 will also be determined)

On the morning of Day 1, urine (first morning void) will be collected for urinary GL-3 and lyso-GB3 determinations followed by administration of the subject's current agalsidase dose given as an infusion using an infusion pump.

Blood samples for pharmacokinetic and pharmacodynamic analysis will be collected immediately before initiation of agalsidase infusion and over the 24-hour period after initiation of the agalsidase infusion. Plasma and WBC α-Gal A enzyme levels, plasma lyso-GB3 and

plasma antibody titer will be determined from the collected blood samples at the times summarized in Table 2 for agalsidase beta and in Table 5 for agalsidase alfa. A 12-lead ECG will be performed at the end of the agalsidase infusion, immediately after collection of the post-infusion blood sample. Vital signs will be recorded as defined in Section 8.4.9. Adverse events and concomitant medications will also be assessed.

On Day 2, a punch skin biopsy will be collected 24 hours after initiation of the previous day's infusion from which α-Gal A enzyme levels will be determined; if sufficient sample is available, skin GL-3 will also be determined. After collection of the last pharmacokinetic sample and before discharge, the following assessments will be performed:

- Adverse event assessment
- Concomitant medications
- Physical examination
- Vital signs
- Weight
- Clinical laboratory tests (serum chemistry, hematology, and urinalysis)
- A blood sample for WBC and plasma α-Gal A levels

On Day 7, subjects will return to the clinical site and will have the following assessments performed:

- Adverse event assessment
- Concomitant medications
- Physical examination
- Vital signs
- Skin biopsy (punch biopsy for measurement of α-Gal A enzyme levels; if sufficient sample is available, skin GL-3 will also be determined)
- A blood sample for WBC and plasma α-Gal A levels, and plasma lyso-GB3 levels

On Day 14, a urine sample (first morning void) for determination of urinary GL-3 excretion and lyso-GB3 will be collected. A blood sample for WBC and plasma α-Gal A levels, and plasma lyso-GB3 levels will also be collected. In addition, the following assessments will be performed:

- Adverse event assessment
- Concomitant medications
- Vital signs

For subjects on a bi-weekly infusion schedule, Day -1 of Period 2 may overlap with Day 14 of Period 1.

8.3.3.2. Period 2

Subjects will be re-admitted to the clinical site approximately 10 hours prior to their next scheduled agalsidase infusion. Subjects will have the following assessments performed at check-in (Day -1):

- Adverse event assessment
- Concomitant medications
- Physical exam

- Vital signs
- Weight
- 12-lead ECG
- Clinical laboratory tests (serum chemistry, hematology, and urinalysis)

On the morning of Day 1, urine (first morning void) will be collected for urinary GL-3 and lyso-GB3 determinations followed by administration of an oral dose of 450 mg of AT1001 2 hours prior to the scheduled agalsidase infusion. Subjects will fast for at least 2 hours before and 2 hours after AT1001 administration. In Period 2, each subject will receive the identical agalsidase dose administered in Period 1 as an infusion using an infusion pump. The agalsidase infusion will be initiated 2 hours after administration of the AT1001 dose.

Blood samples for pharmacokinetic and pharmacodynamic analysis will be collected before dosing of AT1001 and at 1 hour after administration of AT1001. Additional blood samples will be collected immediately before initiation of the agalsidase infusion and over the 24-hour period after initiation of the agalsidase infusion. Plasma and WBC α-Gal A enzyme levels, plasma lyso-GB3 and plasma antibody titer will be determined from the collected blood samples at the times summarized in Table 3 for agalsidase beta and in Table 6 for agalsidase alfa. A 12-lead ECG will be performed at the end of the agalsidase infusion, immediately after collection of the post-infusion blood sample. Vital signs will be recorded as defined in Section 8.4.9. Adverse events and concomitant medications will also be assessed.

On Day 2, a punch skin biopsy will be collected 24 hours after initiation of the infusion from which α-Gal A enzyme levels will be determined; if sufficient sample is available, skin GL-3 will also be determined. A blood sample for WBC and plasma α-Gal A levels, and plasma lyso-GB3 levels will be collected.

After collection of the last pharmacokinetic sample and before discharge, the following assessments will be performed:

- Adverse event assessment
- Concomitant medications
- Physical examination
- Weight
- Vital signs
- Clinical laboratory tests (serum chemistry, hematology, and urinalysis)

On Day 7, subjects will return to the clinical site and have the following assessments performed:

- Adverse event assessment
- Concomitant medications
- Physical examination
- Vital signs
- Skin biopsy (punch biopsy for measurement of α-Gal A enzyme levels; if sufficient sample is available, skin GL-3 will also be determined)
- A blood sample for WBC and plasma α-Gal A levels, and plasma lyso-GB3 levels

On Day 14, a urine sample (first morning void) for determination of urinary GL-3 and lyso-GB3 excretion will be collected. A blood sample for WBC and plasma α-Gal A levels and plasma lyso-GB3 will also be collected. In addition, the following assessments will be performed:

- Adverse event assessment
- Concomitant medications
- Vital signs

8.3.4. Follow-Up Visit

Subjects who complete all periods of each dose level or prematurely discontinue will be asked to return for a safety follow up visit, 1 month after the last treatment or last study visit (whichever is later), during which the following assessments will be performed:

- Physical examination
- Concomitant medication assessment
- Vital signs
- Weight
- 12-lead ECG
- Clinical laboratory tests (hematology, serum chemistry (including eGFR), and urinalysis
- WBC α-Gal A activity, urine GL-3 and lyso-GB3 (assessed using first morning urine)
- Adverse event assessment

8.3.5. Unscheduled Visits

If deemed necessary to ensure subject safety, the investigator can perform an unscheduled visit. If any laboratory assessments, needed immediately for clinical management purposes, are tested locally it is recommended that duplicate samples also be sent for central laboratory analysis. The date and reason for the visit, in addition to information collected from procedures performed, are to be captured in the subject's source notes and CRF.

8.4. Assessments

8.4.1. AT1001 and rhα-Gal A Pharmacokinetics

Blood samples will be collected at the times described in Section 8.3. Concentrations of AT1001 will be measured in plasma using a validated Liquid Chromatography-Tandem Mass Spectrometry (LC-MS/MS) assay. α-Gal A levels in plasma will be determined by a qualified assay measuring enzyme activity using 4-methylumbelliferone glucuronide (4-MUG). α-Gal A protein levels will be measured by Western blotting using anti-human Gal A antibody.

Additional information on collection, processing and shipping procedures will be provided in the laboratory manual.

8.4.2. α-Gal A Enzyme Levels in Skin

α-Gal A enzyme levels will be examined in skin biopsy samples. Skin biopsies will be done using a "punch" device. One piece will be removed at each visit as described on Section 8.3.

α-Gal A levels in skin will be determined by a qualified assay measuring enzyme activity using 4-MUG. α-Gal A protein levels will be measured by Western blotting using anti-human Gal A antibody.

Additional information will be provided in the laboratory manual.

8.4.3. WBC α-Gal A Levels

Blood samples will be collected at the times described in Section 8.3 for determination of WBC α-Gal A enzyme levels. α-Gal A levels in WBCs will be determined by a qualified assay measuring enzyme activity using 4-MUG. WBC α-Gal A protein levels will be measured by Western blotting using anti-human Gal A antibody.

See the laboratory manual for procedures for preparing WBC lysates.

8.4.4. Plasma Lyso-GB3

Plasma lyso-GB3 levels are increased in patients with Fabry disease and appear to be related to the clinical condition of patients receiving ERT. [29-31] Lyso-GB3 may have a role in glomerular injury in Fabry disease by promoting the release of secondary mediators of glomerular injury.[32]

Measurements of plasma lyso-GB3 will be performed on an exploratory basis to obtain data in patients receiving ERT alone and ERT with co-administered AT1001. Blood samples will be collected at the times described in Section 8.3. Concentrations of lyso-GB3 will be measured in plasma using a qualified assay.

8.4.5. Urine GL-3 and Lyso-GB3

The urinary excretion of lyso-GB3 in Fabry patients has been shown to correlate with a number of indicators of disease severity. Lyso-GB3 was not detected in urine from healthy controls.[33]

A first in morning void urine sample will be collected from each subject for analysis of urine GL-3 and lyso-GB3 excretion on Day -1, Day 1 and Day 14 of Periods 1 and 2 as described in Section 8.3. The subjects will collect urine in the morning of Day -1, Day 1, and Day 14. Urinary GL-3 and urinary lyso-GB3 will be expressed as a function of urinary creatinine concentration.

Additional information will be provided in the laboratory manual.

8.4.6. Antibody Titer

Blood samples will be collected at the visits and times described in Section 8.3. Total antibody titers and potentially neutralizing antibody ex-vivo in an in vitro assay may be measured. Additional information will be provided in the laboratory manual.

8.4.7. Sample Handling and Shipment

Sample handling and shipment instructions will be outlined in the laboratory manual.

8.4.8. Safety Parameters

Safety will be assessed by review of changes in physical exam findings, vital signs, ECG changes over time, clinical labs and adverse events. The definitions, reporting and follow-up of AEs and SAEs are described in Section 9.

8.4.9. Vital Signs, Weight and Height

To monitor safety, body temperature, respiration, seated blood pressure and heart rate will be measured at screening and check-in (Day-1), before dosing and approximately 1, 2, 3, 4, and 6 hours following administration of agalsidase (Period 1) and AT1001 (Periods 2 and 3), on Days 2, 7 and 14. Where the time of vital sign monitoring coincides with a blood draw, the blood draw will take precedence and the vital signs will be adjusted accordingly.

Body weight and height will be recorded at times described in Section 8.3.

8.4.10. ECG Monitoring

A standard 12-lead ECG will be performed at the times described in Section 8.3. Significant findings not present prior to start of treatment, which meet the definition of an AE, must be recorded in the CRF. Where the time of the ECG monitoring coincides with a blood draw, the blood draw will take precedence and the time of the ECG monitoring will be adjusted accordingly.

8.4.11. Clinical Laboratory Tests

Blood samples for clinical laboratory tests (hematology, serum chemistry) and urinalyses will be collected at every visit and analyzed at the central laboratory:

- Hematology tests include total hemoglobin, hematocrit, erythrocyte, platelet, and leukocyte counts with differential.
- Coagulation (screening only) includes International Normalized Ratio (INR) and Activated Partial Thromboplastin Time (aPTT).
- Serum chemistry includes measurement of aspartate aminotransferase (AST), alanine aminotransferase (ALT), alkaline phosphatase, total bilirubin, creatinine, urea, glucose, calcium, sodium, potassium, magnesium, total protein, albumin, bicarbonate, LDH, blood urea nitrogen, chloride, and phosphate.
- Urinalysis includes color, appearance, specific gravity, pH, protein, glucose, ketones, blood, leukocyte esterase, nitrite, bilirubin, urobilinogen, and microscopy of sediment.

Measurement of serum creatinine will be performed using reagents that have been calibrated to an isotope dilution mass spectrometry (IDMS) reference method.

The Investigator or his/her designee will review each laboratory report from the central laboratory and assess any out of range laboratory results as "not clinically significant" (NCS) or "clinically significant" (CS). Any results that are outside the laboratory normal range which are considered clinically significant will require a repeat test as soon as possible. This will rule out laboratory analysis error. Persistent clinically significant results that are outside the laboratory

range will be repeated until the analyte returns to normal, or until an etiology is determined. The Investigator (or his/her designee) will sign and date all laboratory reports.

Clinically significant laboratory abnormalities must be reported by the Investigator as an AE or SAE as appropriate.

8.4.12. Adverse Events

Subjects will be monitored throughout the study for adverse reactions to the study formulations and/or procedures.

8.5. General and Dietary Restrictions

Subjects are required to fast 2 hours before and 2 hours after taking each dose of AT1001. It is acceptable for subjects to drink water while fasting. Subjects should otherwise maintain normal food and fluid intake for the duration of the study.

9. ADVERSE EVENTS

9.1. Definitions

9.1.1. Adverse Event

An adverse event is any untoward medical occurrence in a subject administered a pharmaceutical product, biologic (at any dose), or medical device, which does not necessarily have a causal relationship with the treatment. Therefore, an AE can be any unfavorable and unintended sign (including an abnormal laboratory finding), symptom, or disease temporally associated with the use of a medical product, whether or not considered related to the medical product. AEs may include the onset of new illness and the exacerbation of pre-existing conditions.

The routine evolution of the disease condition under treatment according to the protocol will be evaluated as part of the disease symptoms assessments. Changes in the disease condition may not qualify as AEs. Changes in Fabry disease symptoms must be reviewed by the Investigator, or other designated and qualified member of the study staff, and be marked as ‘Clinically Significant’ or ‘Not Clinically Significant’ in the subject’s source notes. If there is a clinically relevant worsening of a sign or symptom of the condition under treatment and the outcome fulfills the definition of an AE, it must be reported as directed in the protocol.

9.1.2. Serious Adverse Event

An adverse event or suspected adverse reaction is considered serious if, in the view of either the investigator or sponsor, it results in any of the following outcomes:

- Death
- Life-threatening adverse event (places the subject at immediate risk of death from the AE as it occurs; it does not refer to an event which hypothetically might have caused death if it were more severe) as assessed by either the investigator or the sponsor
- Inpatient hospitalization or prolongation of existing hospitalization
- Persistent or significant incapacity or substantial disruption of the ability to conduct normal life functions
- Congenital anomaly/birth defect
- Important medical events

An important medical event that may not result in one of the above serious outcomes may be considered an SAE when, based upon appropriate medical judgment, it may jeopardize the subject and may require medical or surgical intervention to prevent one of the serious outcomes. Examples of such medical events include allergic bronchospasm requiring intensive treatment in an emergency room or at home, blood dyscrasias or convulsions that do not results in in-patient hospitalization, or the development of drug dependency or drug abuse.

9.1.3. Relationship to Study Medication

The Investigator or a qualified sub Investigator will review each event and assess its relationship to the study medication based on available information according to the following guidelines:

- Definite: A reaction that follows a distinct temporal relationship from administration of the study medication; that follows a known reaction to the agent or chemical group of the study medication; and that cannot be explained by the subject's clinical state or other factors.
- Probable: A reaction that follows a reasonable temporal sequence from administration of the study medication; that follows a known or expected response pattern to the suspected study medication; and that could not be reasonably explained by the known characteristics of that subject/patient's clinical state.
- Possible: A reaction that follows a reasonable temporal sequence from administration of the study medication; that follows a known or expected response pattern to the suspected study medication; but that could readily have been produced by a number of other factors.
- Unlikely: A reaction that does not follow a reasonable temporal sequence from administration of the study medication. However, causality from the study medication cannot be ruled out.
- Unrelated: A reaction for which sufficient data exist to indicate that the etiology is unrelated to the study medication.

For the purpose of reporting serious adverse events to regulatory authorities and ethics committees, any AE assessed by the principal Investigator as definitely, probably, or possibly related to AT1001 will be considered "related" to study medication (i.e., associated with the use of the study medication). Any AE assessed as unlikely or unrelated will be considered "not related" to study medication (i.e., not associated with the use of study medication).

9.1.4. Assessment of Severity

The following definitions for rating severity will be used:

- Mild: Awareness of sign, symptom or event, but the AE is easily tolerated and does not interfere with daily activity.
- Moderate: Discomfort enough to cause interference with usual activity and may warrant intervention, but the subject is still able to function.
- Severe: Incapacitating with inability to do usual activities or significantly affects clinical status, and requires medical intervention.

When the determination of AE severity rests on medical judgment, the determination of severity must be made with the appropriate involvement of the Investigator or a qualified sub Investigator.

9.2. Reporting of Adverse Events

Information regarding AEs is to be obtained by questioning or examining the subject.

At each visit, beginning from the time written informed consent is provided until 30 days after the last study visit, all new complaints and symptoms (i.e., those not existing before the subject provides informed consent) must be recorded as AEs in the CRF and in the subject's medical records.

- Pre-existing complaints or symptoms that increased in intensity or frequency after the subjects provides informed consent must be entered in the adverse event CRF page.

- Clinically significant laboratory abnormalities must be reported by the Investigator as an AE or SAE as appropriate.

For each AE reported, the date and time the event started and ended, action taken, outcome (resolved, resolved with sequelae, ongoing, or fatal), relationship to the study medication and severity must be noted.

All subjects who have adverse events, whether or not the event is considered associated with the use of AT1001, must be monitored to determine the outcome. The clinical course of the adverse event will be followed according to accepted standards of medical practice, even after the end of the period of observation, until a satisfactory explanation is found or the Investigator considers it medically justifiable to terminate follow-up. Should the adverse event result in death, a full pathologist's report should be supplied, if possible. For all AEs that require or result in subject discontinuation from the study, relevant clinical assessments and laboratory tests will be repeated as clinically appropriate, until final resolution or stabilization of the event(s).

9.3. Additional Reporting Requirements for Serious Adverse Events

If the adverse event is serious (see Section 9.1.2), the Investigator must complete the adverse event section of the CRF, and also submit an SAE report at the time the serious adverse event is detected. All SAEs must be reported by the Investigator to the Sponsor or Sponsor's representative immediately, but no later than within 24 hours of the Investigator's knowledge of the event.

Every attempt should be made to describe the adverse event in terms of a diagnosis. If a clear diagnosis has been made, individual signs and symptoms will not be recorded unless they represent atypical or extreme manifestations of the diagnosis, in which case they should be reported as separate events. If a clear diagnosis cannot be established, each sign and symptom must be recorded individually.

Serious adverse events must be documented and supplied to the Sponsor immediately, but no later than within 24 hours of the investigator's knowledge of the event. SAE reports should be faxed to the attention of medical monitor at Fabry Safety at +1-646-963-2056. The investigator must also inform the study monitor in all cases. The Sponsor will ensure that all regulatory reporting requirements are met.

The initial report must be as complete as possible, including details of the current illness and serious adverse event, and an assessment of the causal relationship between the event and AT1001 or the trial procedure. Information not available at the time of the initial report (e.g., an end date for the adverse event or laboratory values received after the report) must be documented in a follow-up Serious Adverse Event report.

If a non-serious event becomes serious, details must be forwarded immediately, but no later than 24 hours of the investigator's knowledge of the event, to the Sponsor in a Serious Adverse Event report.

If the Investigator detects an SAE in a subject more than 30 days after the end of the period of observation, and considers the event possibly, probably, or definitely related to previous study treatment, s/he should contact the Sponsor to determine how the adverse event should be documented and reported.

The expectedness of an AE shall be determined by the Sponsor, according to the Sponsor's reference document (e.g., the current Investigator Brochure or other safety-related information as available).

9.4. Reporting of Serious Adverse Reactions

Any adverse event that is serious, associated with the use of the study medication, and unexpected (also referred to as a suspected unexpected serious adverse reaction or SUSAR) has additional reporting requirements, as described below.

If the SUSAR is fatal or life-threatening, associated with the use of the study medication, and unexpected, regulatory authorities and ethics committees will be notified within 7 calendar days after the Sponsor learns of the event. Additional follow-up information may be reported within an additional 8 days (15 days total).

If the SUSAR is not fatal or life-threatening but is otherwise serious, associated with the use of the study medication, and unexpected, regulatory authorities and ethics committees will be notified within 15 calendar days after the Sponsor learns of the event.

The Sponsor will notify the Investigators of the SUSAR in accordance with applicable regulations. Follow-up information may be submitted if necessary. Adverse events will be reported to the relevant regulatory agencies according to the rules in effect in each country where study sites are located.

The Sponsor will also provide annual safety updates to the regulatory authorities and ethics committees responsible for the trial. These updates will include information on SUSARs and other relevant safety findings.

9.5. Other Reporting Requirements

9.5.1. Reporting of Pregnancy

Pregnancy in and of itself is not regarded as an AE; however, pregnancy information on study subjects is collected by Amicus. If the female partner of a male subject becomes pregnant during the subject's participating in the study, the Sponsor must be informed within 5 working days of the Investigator or study staff becoming aware of the pregnancy.

9.5.2. Reporting of Overdose

Any event associated with or observed in conjunction with a product overdose (whether accidental or intentional) is considered by the Sponsor to be an AE and must be reported as such. If a subject experiences an overdose (defined as higher than the dose of study medication prescribed in the protocol) during the course of the study (whether symptomatic or not), the Sponsor must be informed within 5 working days of the Investigator or study staff first becoming aware of the overdose. Follow-up information must be forwarded on the outcome as applicable. If an SAE occurs in conjunction with the overdose, then the reporting time frame for an SAE (immediately, but no more than 24 hours) must be met.

9.5.3. Reporting of Possible Study Medication Product Quality Defects

Any defect or possible defect associated with the study medication provided by the Sponsor must be reported by the Investigator or study staff to the Sponsor within 1 working day of first becoming aware of the possible defect. The study medication and packaging components in question, if available, must be stored in a secure area under the specified storage conditions until it is determined whether the study medication and/or packaging is required for investigation of the possible defect. If the possible defect is associated with an SAE, the SAE must be reported as per SAE reporting criteria and timelines above, and the SAE report must mention the possible study medication defect complaint.

10. WITHDRAWALS

10.1. Withdrawal of Subjects

Subjects may discontinue study medication or be withdrawn from the study for the following reasons:

- At their own request or at the request of their parent or guardian (if the subject is a minor) or legally authorized representative
- If, in the Investigator's opinion, continuation in the study would be detrimental to the subject's well-being
- Occurrence of an intolerable treatment-emergent adverse event as determined by the Investigator and/or the subject
- Failure of the subject to return to the study site for scheduled visits
- Persistent noncompliance

In all cases, the reason for and date of withdrawal must be recorded in the CRF and in the subject's medical records. The subject must be followed up to establish whether the reason was an adverse event, and, if so, this must be reported.

In case the subject wishes to discontinue treatment, the Investigator must inquire and document in the medical record whether:

- the subject only wants to discontinue study medication, but agrees to the follow-up procedures as outlined in the protocol
- the subject wants to discontinue study medication and all follow-up procedures
- the subject wants to revoke the consent to collect and use further data

Note: In the United States, the authorization to use and disclose data for research can only be revoked in writing by the subject.

The Investigator must make every effort to contact subjects who discontinue study medication or visits or are lost to follow-up and schedule the end of treatment (EOT) assessments (see Section 10.1.4.). Attempts to contact such subjects must be documented in the subject's records (e.g., times and dates of attempted telephone contact, receipt for sending a registered letter).

10.2. Replacement of Subjects

Subjects who withdraw from the study will be replaced. Subjects may repeat a treatment period under certain exceptional circumstances such as changes in their infusion rates or durations, which make their data non-evaluable. Consultation with the Amicus medical monitor is required prior to any such re-challenge. Subjects who enroll in Stage 1 may be re-enrolled for Stage 2 if they provide written consent and agree to all study procedures. A washout period of at least 30 days post-migalastat HCl will be required prior to the first ERT dosing in Stage 2.

11. EMERGENCY PROCEDURES

11.1. Emergency Sponsor Contact

In emergency situations, the Investigator should contact the Sponsor by telephone at the number given on the title page of the protocol.

11.2. Emergency Treatment

During and after a subject's participation in the trial, the Investigator or institution should ensure that adequate medical care is provided to a subject for any adverse events, including clinically significant laboratory values, related to the trial. The Investigator or institution should inform a subject when medical care is needed for intercurrent illness(es) of which the Investigator becomes aware.

12. DATA ANALYSIS

12.1. Analysis Populations

The population for pharmacokinetic analysis will include all subjects who have successfully completed all three periods in Stage 1 or both periods in Stage 2.

The safety population will include all enrolled subjects who receive at least one dose of agalsidase or AT1001. All safety analyses will be performed using the safety population and will analyze subjects according to the treatment(s) received.

12.2. Primary Endpoints

12.2.1. Pharmacokinetic Parameters

Non-compartmental pharmacokinetic parameters of $AUC_{0\text{-}t}$, $AUC_{0-\infty}$, Cmax, t_{max}, k_{el} and half life will be calculated from plasma AT1001 and plasma rhα-Gal A concentrations. Pharmacokinetic parameters will be summarized by treatment using descriptive statistics. The $AUC_{0\text{-}t}$, $AUC_{0-\infty}$, ratios for each compound alone to the respective compound in combination will be calculated.

12.2.1.1. Statistical Procedures

Descriptive statistics (N, mean, geometric mean, standard deviation, and coefficient of variation, standard error, median, minimum and maximum) will be provided as appropriate. The effect of a compound on the co-administered compound will be evaluated by calculation of the individual (by subject) AUC and C_{max} ratios as follows:

$$\text{AUC Ratio} = \frac{AUC_{infinity\ (combination)}}{AUC_{infinity(alone)}}$$

$$C_{max}\ \text{Ratio} = \frac{C_{max\ (combination)}}{C_{max(alone)}}$$

The AUC and C_{max} ratios will be expressed as a mean of the individual ratios and 90% confidence interval for the mean.

Results will be presented in tabular and graphic forms, as appropriate. All subjects who are dosed with study medication and have sufficient data to generate reliable pharmacokinetic parameters will be included in the safety and pharmacokinetic analysis.

12.2.1.2. Statistical Analysis

Additional statistical analyses, other than those described in this section, may be performed if deemed appropriate.

12.2.2. Safety Evaluation (Primary Endpoint)

Safety parameters will be summarized using descriptive statistics. Frequency and percentages will be provided for categorical variables. Sample size (n), mean, standard deviation, quartiles, minimum, and maximum will be provided for continuous variables. Where relevant, changes from baseline will be presented. In addition, shift analysis (e.g., a table summarizing changes from normal to out-of-normal range) may be provided for selected clinical laboratory results.

The frequency and proportion of clinically notable laboratory values will be summarized overall and for each study visit through Month 6 by group. Physical examination results will be categorized as normal, abnormal clinically significant, or abnormal not clinically significant, and summarized for each study visit, through Month 6, and where relevant; a normal-to-abnormal change table will be provided.

Adverse events (AEs) will be coded using the Medical Dictionary for Regulatory Activities (MedDRA) dictionary. The number and percent of subjects reporting each AE will be summarized and presented by system organ class (SOC) and preferred term (PT) for each group. A by-subject AE data listing including verbatim term, SOC, PT, treatment group, severity, and relationship to treatment will be provided. The incidence of treatment emergent AEs will be summarized and presented by MedDRA class and severity for treatment group. AEs with an incidence of at least 5% in either group will be compared using the Fisher exact test.

Concomitant medications will be coded using World Health Organization (WHO) Drug dictionary and summarized by Anatomical Therapeutic Chemical (ATC), preferred term and treatment group. All concomitant medications and medical histories will be listed by subject.

Additional details of the statistical analysis of the safety endpoints are provided in the statistical analysis plan.

12.3. Secondary Endpoint

12.3.1. Skin α-Gal A Levels

Descriptive statistics (N, mean, geometric mean, standard deviation, and coefficient of variation, standard error, median, minimum, and maximum) for enzyme levels in skin biopsy samples will be provided as appropriate. The effect of AT1001 on the enzyme levels in the skin sample will be evaluated by appropriate statistical techniques.

12.4. Interim Analysis

Interim analysis may be performed as needed.

12.5. Sample Size Calculation

The number of subjects chosen was considered appropriate to meet the objectives of the study.

13. ETHICAL AND LEGAL ASPECTS

13.1. Good Clinical Practice

This study is to be conducted according to globally accepted standards of good clinical practice (as defined in the ICH E6 Guideline for Good Clinical Practice, 01 May 1996), in agreement with the Declaration of Helsinki and in keeping with local regulations.

13.2. Delegation of Investigator Duties

The Investigator should ensure that all persons assisting with the trial are adequately qualified, informed about the protocol, any amendments to the protocol, the study treatments, and their trial-related duties and functions.

The Investigator should maintain a list of sub Investigators and other appropriately qualified persons to whom he or she has delegated significant trial-related duties.

Should the Investigator delegate the supervision of AT1001 administration to a designated person, this individual must have the appropriate medical qualifications to effectively conduct or supervise any potential resuscitation procedures.

Even if the Investigator delegates any trial-related duties to other qualified members of his or her study staff, the Investigator retains ultimate responsibility for obtaining informed consent from study subjects; for ensuring that the investigation is conducted according to the protocol, the signed investigator statement (Form FDA 1572), and applicable regulations; for protecting the rights, safety, and welfare of study subjects; and for the control of Investigational Products under evaluation.

13.3. Subject Information and Informed Consent

Before being enrolled in the clinical study, subjects must consent to participate after the nature, scope, and possible consequences of the clinical study have been explained in a form understandable to them and all of their questions regarding the study have been answered.

An informed consent document that includes both information about the study and the consent form will be prepared and given to the subject. This document will contain all the elements required by the ICH E6 Guideline for Good Clinical Practice and any additional elements required by local regulations. The document must be in a language understandable to the subject and must specify who informed the subject. Where required by local law, the person who informs the subject must be a physician.

After reading the informed consent document, the subject must give consent in writing. The subject's consent must be confirmed at the time of consent by the personally dated signature of the subject and by the personally dated signature of the person conducting the informed consent discussions. A copy of the informed consent will be given to the subject and the original will be retained by the site. An entry must be made in the subject’s dated source documents to confirm that informed consent was obtained prior to any study-related procedures and that the subject received a copy.

If the subject is unable to read, oral presentation and explanation of the written informed consent form and information to be supplied to subjects must take place in the presence of an impartial witness. Consent must be confirmed at the time of consent orally and by the personally dated signature of the subject or by a local legally recognized alternative (e.g., the subject's thumbprint or mark). The witness and the person conducting the informed consent discussions must also sign and personally date the consent document. Details about why oral presentation was used, how the information was presented, and how the subject provided consent must be described in the medical record.

The Investigator will not undertake any measures specifically required only for the clinical study until written consent has been obtained.

The Investigator may inform the subject’s primary physician about the subject’s participation in the trial if the subject has a primary physician and if the subject agrees to the primary physician being informed.

13.4. Confidentiality

Subject names will not be supplied to the Sponsor. A subject number will be recorded in the CRF, and if the subject name appears on any other document (e.g., laboratory report), it must be obliterated on the copy of the document to be supplied to the Sponsor. Study findings stored on a computer will be stored in accordance with local data protection laws. The subjects will be informed that representatives of the Sponsor, IEC/IRB, or regulatory authorities may inspect their medical records to verify the information collected, and that all personal information made available for inspection will be handled in strictest confidence and in accordance with local data protection laws.

The Investigator will maintain a personal subject identification list (subject numbers with the corresponding subject names) to enable records to be identified.

13.5. Protocol Amendments

Any non-administrative changes to the protocol initiated either by the Sponsor or the Investigator requires a formal amendment procedure. Approval of all amendments must be obtained from the Sponsor, Investigator, Regulatory Authorities (if applicable), and IEC/IRB prior to implementation.

Any protocol deviation or change intended to eliminate an apparent immediate hazard to a subject may be implemented immediately, as described in 21 CFR 312.30(b) (2). The Sponsor and IEC/IRB must be notified within 1 business day.

13.6. Approval of the Clinical Study Protocol and Amendments

Before the start of the study, the clinical study protocol, informed consent document, and any other appropriate documents will be submitted to the IEC/IRB for approval. The documents will also be submitted to the regulatory authorities, in accordance with local requirements.

Study medication can only be supplied to the Investigator after documentation of all ethical and regulatory requirements for starting the study has been received by the Sponsor. This documentation must also include an IRB membership list or other documentation confirming the

IEC/IRB's compliance with federal or other applicable regulations. If the IEC/IRB will not disclose the names of the committee members, it should be asked to issue a statement confirming that the composition of the committee is in accordance with GCP. Formal approval by the IEC/IRB should preferably mention the study title, protocol number, study site (or region or area of jurisdiction, as applicable), amendment number where applicable, and any other documents reviewed. It must mention the date on which the decision was made and must be officially signed by a committee member.

Before the first subject is enrolled in the study, all ethical and regulatory requirements must be met.

The IEC/IRB and, if applicable, the authorities must be informed of all subsequent protocol amendments and administrative changes, in accordance with local regulatory requirements. Amendments must be evaluated to determine whether formal approval must be sought and whether the informed consent document should also be revised.

The Investigator must keep a record of all communication with the IEC/IRB and any communication between the Investigator and the authorities.

13.7. Ongoing Information for IEC/IRB

Unless otherwise instructed by the IEC/IRB, or local law, the Sponsor or the Investigator must submit to the IEC/IRB:

- Information on adverse events that are serious AND unexpected AND associated with AT1001 from the Investigator's site, as soon as possible
- Expedited safety reports from the Sponsor, as soon as possible
- Periodic reports on the progress of the study

13.8. Closure of the Study

The study must be closed at the site on completion.

Completion or premature termination of the study will be reported by the Sponsor to the regulatory agency and by the Sponsor or by the Investigator to the IEC/IRB as required by local regulation or by the IEC/IRB.

Furthermore, the Sponsor or the Investigator has the right to close this study site at any time. As far as possible, premature closure should occur after mutual consultation.

Study materials must be returned, disposed of, or retained as directed by the Sponsor.

13.9. Record Retention

The Investigator must obtain approval in writing from the Sponsor before destruction of any records.

Essential documents should be retained until at least 2 years after the last approval of a marketing application in an ICH region and until there are no pending or contemplated marketing applications in an ICH region, or at least 2 years have elapsed since the formal discontinuation of clinical development of AT1001. However, because of international regulatory requirements or country-specific requirements, the Sponsor may request retention for a longer period.

Prior to any decision regarding the disposal or destruction of study documents, the Investigator should contact the Sponsor to inform them. The Sponsor may request that the site take alternative actions other than disposal or destruction of study documents.

Essential documents include:

- Signed informed consent documents for all subjects
- Subject identification code list, screening log (if applicable) and enrollment log
- Record of all communications between the Investigator and the IEC/IRB
- Composition of the IEC/IRB (or other applicable statement as described in Section 12.6)
- Record of all communications between the Investigator and Sponsor (or its designee)
- List of sub Investigators and other appropriately qualified persons to whom the Investigator has delegated significant trial-related duties, together with their roles in the study and their signatures
- Copies of CRFs and of documentation of corrections for all subjects
- Investigational product accountability records
- Record of any body fluids or tissue samples retained
- All other source documents (subject medical records, hospital records, laboratory records, etc.)
- All other documents as listed in section 8 of the ICH E6 Guideline for Good Clinical Practice (Essential Documents for the Conduct of a Clinical Trial)

Normally, these records will be held in the Investigator's archives. If the Investigator is unable to meet this obligation, he or she must ask the Sponsor for permission to make alternative arrangements. Details of these arrangements should be documented.

13.10. Liability and Insurance

Liability and insurance provisions for this study are given in separate agreements.

13.11. Financial Disclosure

Before the start of the study, the Investigator will disclose to the Sponsor any proprietary or financial interests he or she might hold in the investigational products or the Sponsor company as outlined in the financial disclosure form provided by the Sponsor. The Investigator agrees to update this information in case of significant changes during the study or within 1 year of its completion. The Investigator also agrees that, where required by law or regulation, the Sponsor may submit this financial information to domestic or foreign regulatory authorities in applications for marketing authorizations.

Where required by regulation the Investigator or Sponsor on behalf of the Investigator will also disclose these financial interests to the IEC/IRB and the Investigator will disclose his/her financial interests to the subjects in the informed consent information.

Where required by regulation the Sponsor or Investigator will also submit the financial arrangements for the study to the regulatory authorities or to the IEC/IRB.

Financial disclosures will be provided by each sub Investigator to whom the Investigator delegates significant study related responsibilities.

13.12. Protocol Adherence

Adherence to the protocol is required. Any significant changes to the clinical trial will be written into a protocol amendment and submitted to the IEC/IRB (and, where applicable, regulatory authorities) for approval before implementation of the change(s).

Protocol deviations to inclusion/exclusion criteria, addition or deletion of tests, dosing and/or duration of treatment or any other aspect of the study design that may significantly impact subject safety or erode data/scientific integrity, are not permitted under Good Clinical Practice or by the Sponsor unless necessary to eliminate an immediate hazard to the subject (or subjects). Where the Sponsor and/or Investigator must take urgent safety measures to protect trial subjects from an immediate hazard, a protocol deviation may be allowed before obtaining approval from the relevant IEC/IRB (and/or regulatory authorities). In such cases, the IEC/IRB (and/or regulatory authorities) shall be notified of the need for the urgent protocol deviation, the measures taken, and the plan for further action. In all cases, reporting to IEC/IRBs and regulatory authorities will comply with applicable local requirements.

The Sponsor and IEC/IRB, where required by local law, must be informed of all deviations/violations from the protocol and the Investigators shall document such protocol deviations/violations in subject source records and CRFs.

14. STUDY MONITORING AND AUDITING

Monitoring and auditing procedures developed or endorsed by the Sponsor will be followed, in compliance with GCP guidelines. Direct access to the on-site study documentation and medical records must be ensured.

14.1. Study Monitoring and Source Data Verification

Monitoring will be done by personal visits from a representative of the Sponsor (study monitor) who will check the CRFs for completeness and clarity, and crosscheck them with source documents. In addition to the monitoring visits, frequent communications (letter, e-mail, telephone, and fax) by the study monitor will ensure that the investigation is conducted according to protocol design and regulatory requirements.

Before an investigational site can enter a patient into the study, a representative of the sponsor will visit the investigational study site to:

- Determine the adequacy of the facilities
- Discuss with the investigator(s) and other personnel their responsibilities with regard to protocol adherence, and the responsibilities of the sponsor or its representatives. This will be documented in a Clinical Study Agreement between the sponsor and the investigator.

During the study, a monitor from the sponsor or its representative will have regular contacts with the investigational site, for the following:

- Provide information and support to the investigator(s)
- Confirm that facilities remain acceptable
- Confirm that the investigational team is adhering to the protocol, that data are being accurately recorded in the CRFs, and that investigational product accountability checks are being performed
- Perform source data verification. This includes a comparison of the data in the CRFs with the patient’s medical records at the hospital or practice, and other records relevant to the study. This will require direct access to all original records for each patient (e.g. clinic charts).
- Record and report any protocol deviations not previously sent to the sponsor.
- Confirm AEs and SAEs have been properly documented on CRFs and confirm any SAEs have been forwarded to the sponsor and those SAEs that met criteria for reporting have been forwarded to the IEC/IRB.

The monitor will be available between visits if the investigator(s) or other staff needs information or advice.

The sponsor will be allowed to conduct site visits to the investigational facilities for the purpose of monitoring any aspect of the study. The Investigator agrees to allow the monitor to inspect

the drug storage area, study drug stocks, drug accountability records, subject charts and study source documents, and other records relative to study conduct.

Study close-out will be performed by the study monitor upon closure of the study.

14.2. On-site Audits

Authorized representatives of the sponsor, a regulatory authority, or an IEC/IRB may visit the site to perform audits or inspections, including source data verification. The purpose of a sponsor audit or inspection is to systematically and independently examine all study-related activities and documents to determine whether these activities were conducted, and data were recorded, analyzed, and accurately reported according to the protocol, GCP guidelines of the ICH, and any applicable regulatory requirements. The investigator should contact the sponsor immediately if contacted by a regulatory agency or any other party about an inspection for this study. The investigator should notify the sponsor of inspections regarding other studies, or non-research inspections in a timely fashion.

Domestic and foreign regulatory authorities, the IEC/IRB, and an auditor authorized by the Sponsor may request access to all source documents, CRFs, and other study documentation for on-site audit or inspection. Direct access to these documents must be guaranteed by the Investigator, who must provide support at all times for these activities. Medical records and other study documents may be copied during audit or inspection provided that subject names are obliterated on the copies to ensure confidentiality.

15. DOCUMENTATION AND USE OF STUDY FINDINGS

15.1. Documentation of Study Findings

A CRF will be provided for each subject.

All protocol-required information collected during the study must be entered by the Investigator, or designated representative, in the CRF. Details of CRF completion and correction will be explained to the Investigator. If the Investigator authorizes other persons to make entries in the CRF, the names, positions, signatures, and initials of these persons must be supplied to the Sponsor.

The Investigator, or designated representative, should complete the CRF pages as soon as possible after information is collected, preferably on the same day that a study subject is seen for an examination, treatment, or any other study procedure. Any outstanding entries must be completed immediately after the final examination. An explanation should be given for all missing data.

A source data location list will be prepared prior to study start. This list will be filed in both the trial master file and the Investigator study file and updated as necessary.

The completed CRF must be reviewed and signed by the Investigator named in the clinical study protocol or by a designated sub Investigator.

The Sponsor will retain the originals of all CRFs. The Investigator will retain a copy of all completed CRF pages.

15.2. Use of Study Findings

All information concerning the product as well as any matter concerning the operation of the Sponsor, such as clinical indications for the drug, its formula, methods of manufacture, and other scientific data relating to it, that have been provided by the Sponsor and are unpublished, are confidential and must remain the sole property of the Sponsor. The Investigator will agree to use the information only for the purposes of carrying out this study and for no other purpose unless prior written permission from the Sponsor is obtained.

The Sponsor has full ownership of the original CRFs completed as part of the study.

By signing the clinical study protocol and the confidentiality agreement, the Investigator agrees that the results of the study may be used for the purposes of national and international registration, publication, and information for medical and pharmaceutical professionals. The authorities will be notified of the Investigator's name, address, qualifications, and extent of involvement.

The Sponsor will ensure that a final report on the study is prepared. The Sponsor will ensure that the study findings are reported in a manner that complies with applicable requirements for reporting clinical trial results.

As required by local regulation or by the IRB/IEC, a summary of the clinical study will be submitted by the Sponsor to the regulatory authorities and by the Investigator to the IRB/IEC.

All materials, documents and information supplied by the Sponsor to the Investigator, and all materials, documents and information prepared or developed in the course of the study to be performed under this protocol, shall be the sole and exclusive property of the Sponsor. Subject to obligations of confidentiality, the Investigator reserves the right to publish only the results of the work performed pursuant to this protocol, provided, however, that the Investigator provides an authorized representative of the Sponsor with a copy of any proposed publication for review and comment at least 45 days in advance of its submission for publication. In addition, if requested, the Investigator will withhold publication an additional 90 days to allow for filing a patent application or taking such other measures as Sponsor deems appropriate to establish and preserve its proprietary rights.

It is agreed that, consistent with scientific standards, publication of the results of the study shall be made only as part of a publication of the results obtained by all sites performing the protocol.

Authorship for publications derived from this study will be based on all of the following:
1) substantial contributions to conception and design, or acquisition of data, or analysis and interpretation of data; 2) drafting the article or revising it critically for important intellectual content; and 3) final approval of the version to be published.

16. REFERENCES

1. α-Galactosidase A deficiency: Fabry disease. McGraw-Hill, 2001. (Accessed May 9, 2006, at www.ommbid.com.)
2. Meikle PJ, Hopwood JJ, Clague AE, Carey WF. Prevalence of lysosomal storage disorders. Jama 1999;281:249-54.
3. Spada M, Pagliardini S, Yasuda M, et al. High incidence of later-onset fabry disease revealed by newborn screening. Am J Hum Genet 2006;79:31-40 Epub 2006 Apr 28.
4. Sheth KJ, Roth DA, Adams MB. Early renal failure in Fabry's disease. Am J Kidney Dis 1983;2:651-4.
5. Alroy J, Sabnis S, Kopp JB. Renal Pathology in Fabry Disease. J Am Soc Nephrol 2002;13:S134-8.
6. Becker AE, Schoorl R, Balk AG, van der Heide RM. Cardiac manifestations of Fabry's disease. Report of a case with mitral insufficiency and electrocardiographic evidence of myocardial infarction. Am J Cardiol 1975;36:829-35.
7. Ferrans VJ, Hibbs RG, Burda CD. The heart in Fabry's disease. A histochemical and electron microscopic study. Am J Cardiol 1969;24:95-110.
8. Mendez MF, Stanley TM, Medel NM, Li Z, Tedesco DT. The vascular dementia of Fabry's disease. Dement Geriatr Cogn Disord 1997;8:252-7.
9. Stenson PD, Ball EV, Mort M, et al. Human Gene Mutation Database (HGMD®): 2003 update. Hum Mutat 2003;21:577-81.
10. Askari H, Kaneski CR, Semino-Mora C, et al. Cellular and tissue localization of globotriaosylceramide in Fabry disease. Virchows Arch 2007;451:823-34.
11. Brady OR, Gal, A. E., Bradley, R. M., Martensson, E., Warshaw, A. L., and Laster, L. Enzymatic defect in Fabry's disease: Ceramidetrihexosidase deficiency. N Engl J Med 1967;276:1163-7.
12. Fan J-Q, Ishii S. Active-site-specific chaperone therapy for Fabry disease. Yin and Yang of enzyme inhibitors. FEBS Journal 2007;274:4962-71.
13. Fan J-Q, Ishii S. Cell-based screening of active-site specific chaperone for the treatment of Fabry disease. Methods Enzymol 2003;363:412-20.
14. Asano N, Nash RJ, Molyneux RJ, Fleet GWJ. Sugar-mimic glycosidase inhibitors: natural occurrence, biological activity and prospects for therapeutic application. Tetrahedron: Asymmetry 2000;11:1645-80.
15. Yam GH, Zuber C, Roth J. A synthetic chaperone corrects the trafficking defect and disease phenotype in a protein misfolding disorder. FASEB J 2005;19:12-8.
16. Shin S-H, Murray GJ, Kluepfel-Stahl S, et al. Screening for pharmacological chaperones in Fabry disease. Biochem Biophys Res Commun 2007;359:168-73.
17. Benjamin E, Flanagan J, Schilling A, et al. The pharmacological chaperone 1-deoxygalactonojirimycin increases α-galactosidase A levels in Fabry patient cell lines. J Inherit Metab Dis 2009;32:424-40.
18. Eng CM, Banikazemi M, Gordon RE, et al. A phase 1/2 clinical trial of enzyme replacement in fabry disease: pharmacokinetic, substrate clearance, and safety studies. Am J Hum Genet 2001;68:711-22 Epub 2001 Feb 1.
19. Eng CM, Guffon N, Wilcox WR, et al. Safety and efficacy of recombinant human alpha-galactosidase A-- replacement therapy in Fabry's disease. N Engl J Med 2001;345:9-16.

20. Schiffmann R, Kopp JB, Austin Iii HA, et al. Enzyme Replacement Therapy in Fabry Disease: A Randomized Controlled Trial. JAMA 2001;285:2743-9.
21. West M, Nicholls K, Mehta A, et al. Agalsidase Alfa and Kidney Dysfunction in Fabry Disease. J Am Soc Nephrol 2009;20:1132-9.
22. Replagal™ Physician Prescribing Information (SMPC). http://wwwmedicinesorguk/EMC/printfriendlydocumentaspx?documentid=19760&companyid=1634 2010.
23. Clarke JT, West ML, Bultas J, Schiffmann R. The pharmacology of multiple regimens of agalsidase alfa enzyme replacement therapy for Fabry disease. Genet Med 2007;9:504-9.
24. Genzyme. Fabrazyme® Prescribing Information. In; 2008.
25. Advisory Committee Briefing Document on BLA STN 103979/0: Recombinant human α-Galactosidase for Fabry's disease. 2002. (Accessed February 13, 2008, at http://www.fda.gov/ohrms/dockets/ac/03/briefing/3917B1_03_FDA%20backgrounder.pdf.)
26. Linthorst GE, Hollak CEM, Donker-Koopman WE, Strijland A, Aerts JMFG. Enzyme therapy for Fabry disease: Neutralizing antibodies toward agalsidase alpha and beta. Kidney Int 2004;66:1589-95.
27. Shen J-S, Edwards NJ, Hong YB, Murray GJ. Isofagomine increases lysosomal delivery of exogenous glucocerebrosidase. Biochem Biophys Res Commun 2008;369:1071-5
28. Porto C, Cardone M, Fontana F, et al. The Pharmacological Chaperone N-butyldeoxynojirimycin Enhances Enzyme Replacement Therapy in Pompe Disease Fibroblasts. Mol Ther 2009;17:964–71.
29. Togawa T, Kodama T, Suzuki T, et al. Plasma globotriaosylsphingosine as a biomarker of Fabry disease. Mol Genet Metab 2010;100:257-61
30. Aerts JM, Groener JE, Kuiper S, et al. Elevated globotriaosylsphingosine is a hallmark of Fabry disease. Proceedings of the National Academy of Sciences 2008;105:2812-7.
31. Togawa T, Kawashima I, Kodama T, et al. Tissue and plasma globotriaosylsphingosine could be a biomarker for assessing enzyme replacement therapy for Fabry disease. Biochem Biophys Res Commun;In Press, Accepted Manuscript.
32. Sanchez-Nino MD, Sanz AB, Carrasco S, et al. Globotriaosylsphingosine actions on human glomerular podocytes: implications for Fabry nephropathy. Nephrol Dial Transplant:gfq306.
33. Auray-Blais C, Ntwari A, Clarke JTR, et al. How well does urinary lyso-Gb3 function as a biomarker in Fabry disease? Clin Chim Acta;In Press, Accepted Manuscript.
