# Supplementary material for: Oral Migalastat HCl Leads to Greater Systemic Exposure and Tissue Levels of Active α-Galactosidase A in Fabry Patients when Co-Administered with Infused Agalsidase
Source: PLoS One. 2015 Aug 7;10(8):e0134341. doi: 10.1371/journal.pone.0134341 (PMC4529213; doi:10.1371/journal.pone.0134341)
Supplement: S1 Table — includes the GLA genotype for each Fabry patient enrolled in the study. Most (18 of 23) Fabry patients had non-amenable mutations. (DOCX) [file pone.0134341.s004.docx]

# Supplemental Table S1. *GLA* Genotypes

| ***GLA* GENOTYPE** | | | |
| --- | --- | --- | --- |
| **SubjID** | **Nucleotide Change** | **Amino Acid Change** | **Amenable** |
| AB1.0-150-1 | c.892A>G | N298S | Yes |
| AB1.0-150-2 | c.717delAA | L243GfsX4 | No |
| AB1.0-150-3 | c.35-47del | C12F*fsX*104 | No |
| AB0.5-150-4 | c.748C>T | Q250X | No |
| AB0.5-150-5 | c.661C>T | Q221X | No |
| AB0.5-150-6 | c.708G>A | W236X | No |
| AB0.5-150-7 | c.679C>T | R227X | No |
| AB0.5-150-8 | c.679C>T | R227X | No |
| AA0.2-150-9 | c.44C>A | PALA15GLU | No |
| AA0.2-150-10 | Unknown | P. H125L / P. G128E | No |
| AA0.2-150-11 | Unknown | P H125L / P.G128E | No |
| AA0.2-150-12 | c.1042 G>C | A348P | No |
| AB1.0-450-13 | c.748C>T | Q250X | No |
| AB1.0-450-14 | Unknown | P205T | Yes |
| AB1.0-450-15 | c.679C>T | R227X | No |
| AB1.0-450-16 | c.679C>T | R227X | No |
| AB1.0-450-17 | Unknown | E48Q | Yes |
| AB1.0-150-18 | Unknown | L120P / A121T | No |
| AB0.5-450-19 | c.661C>T | Q221X | No |
| AA0.2-450-20 | c.297-300 DEL AAGA | p.99fsx14 | No |
| AA0.2-450-21 | Unknown | M284T | Yes |
| AA0.2-450-22 | c.1042 G>C | A348P | No |
| AA0.2-450-23 | c.1241 T>C | L414S | Yes |
