# Supplementary material for: Oral Migalastat HCl Leads to Greater Systemic Exposure and Tissue Levels of Active α-Galactosidase A in Fabry Patients when Co-Administered with Infused Agalsidase
Source: PLoS One. 2015 Aug 7;10(8):e0134341. doi: 10.1371/journal.pone.0134341 (PMC4529213; doi:10.1371/journal.pone.0134341)
Supplement: S2 Table — provides mean (CV%) changes from baseline in α-Gal A activity in PBMCs for ERT administered alone and when co-administered with migalastat HCl. Generally, the largest changes from baseline occurred from 2 to 4 hours post-dose during co-administration with migalastat HCl. (DOCX) [file pone.0134341.s005.docx]

| **Supplemental Table S2. Summary of Total α-Gal A Activity Levels in PBMCs** |
| --- |
